# Impact of the physico-chemical properties of surface-initiated polymer brushes on siRNA binding and transfection efficiency

**DOI:** 10.1039/d6bm00813e

**Published:** 2026-09-22

**Authors:** Carlos E. Neri-Cruz, Davide Carta, Julien E. Gautrot

**Affiliations:** a School of Engineering and Materials Science, Queen Mary University of London Mile End Road London E1 4NS UK j.gautrot@qmul.ac.uk

## Abstract

Rational design of polycationic gene delivery vectors requires understanding how polymer chemistry governs RNA complexation, cellular uptake, and intracellular release kinetics. Poly(dimethylaminoethyl methacrylate) brushes were found to display striking potential for the delivery of siRNA, with high knockdown efficiency and low cytotoxicity. However, the impact of the physico-chemistry of a broader range of polymer brushes on RNA delivery has not been explored. Using a library of 10 cationic polymer brushes spanning tertiary amine and quaternary ammonium chemistries, we reveal how hydrophobicity, p*K*_a_, rigidity, and quaternisation modulate siRNA delivery and gene silencing. Combining ellipsometry and surface plasmon resonance, we find that RNA binding capacity correlates directly with pH-responsiveness and the chain rigidity of brushes. Quaternisation with short methyl groups fundamentally altered binding mechanisms, eliminating pH sensitivity while improving colloidal stability and reducing cytotoxicity. To investigate the impact of the physico-chemical properties on transfection efficiency, the uptake of brush-RNA complexes was examined by confocal microscopy and flow cytometry and we quantified cytosolic release using a competitive binding microscopic assay. We observe excellent correlation between the impact of the brush physico-chemistry on release kinetics and long term silencing efficiency. Remarkably, we report that the binding capacity of RNA within brushes does not always correlate with cellular internalisation, competitive desorption and transfection efficiency: rigid imidazole brushes achieved robust gene silencing despite binding substantially less RNA than poly(alkylamine methacrylate) brushes. These findings expand the delivery vector design space, enabling the rational engineering of next-generation polymeric carriers for controlled RNA delivery kinetics.

## Abstract 2

Rational design of polycationic gene delivery vectors requires understanding how polymer chemistry governs RNA complexation, cellular uptake, intracellular release and, in turn, transfection efficiency kinetics.

## Graphical abstract

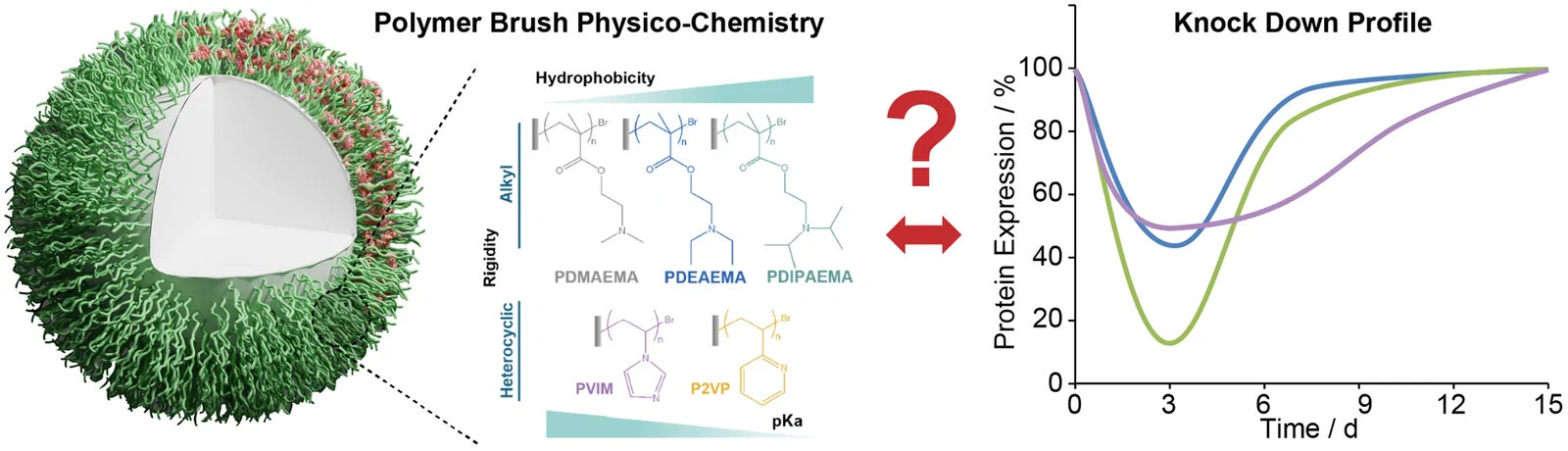

## Introduction

Advancements in gene therapy have unlocked unprecedented opportunities for the precise and programmable modulation of gene function through the delivery of diverse classes of nucleic acid therapeutics. These include small non-coding RNAs such as small interfering RNA (siRNA) and microRNA (miRNA), which mediate sequence-specific gene silencing through the RNA interference (RNAi) pathways,^1–3^ as well as larger genetic constructs, including messenger RNA (mRNA) for transient protein expression,^4^ plasmid DNA (pDNA) for sustained transgene expression and gene-editing systems such as CRISPR-Cas9, enabling targeted and permanent genomic modifications.^5,6^

siRNA technologies are particularly promising for the treatment of genetic diseases, as they selectively target disease-causing genes and downregulate their expression *via* incorporation into the RNA-induced silencing complex (RISC), leading to sequence-specific mRNA degradation.^1–3^ Their rapid clinical progress since 2018 has resulted in eight approved siRNA-based drugs as of 2026. Of these, seven are based on *N*-acetylgalactosamine (GalNAc)-conjugated siRNAs (*e.g.*, GIVLAARI for the treatment of acute hepatic porphyria), whereas only ONPATTRO, indicated for hereditary transthyretin-mediated amyloidosis, employs a lipid-based delivery vector.^1,3,7^ Notably, all currently approved siRNA therapeutics target the liver.^1,3,7^

Despite these advances, achieving efficient and controlled RNA delivery at defined doses, times, and with high specificity and to a variety of organs others than the liver remains challenging. To a large extent, this reflects the inherent complexity of RNA delivery, which requires overcoming multiple biological barriers, including susceptibility to enzymatic degradation, non-specific interactions with other biomacromolecules (leading to loss of functionality and impaired cell/tissue targeting), inefficient cellular uptake, limited endosomal escape, and insufficient intracellular release and engagement with the targeted machinery, all while maintaining an acceptable safety profile and tolerating repeated administration at intervals ranging from weeks to months.^1,3,5,8,9^ The gap between the limited number of siRNA technologies reaching clinical translation compared to the large number that fail during development underscores the need for improved understanding of gene delivery mechanisms.^9,10^

Besides chemical modification of siRNA to enhance stability and reduce off-target effects,^1,3^ siRNA technologies can use delivery vectors to further protect payload integrity while providing additional control over release kinetics and tissue targeting.^5,8,10,11^ Non-viral delivery vectors offering reduced immunogenicity relative to viral vectors have been extensively explored for this purpose, including lipid and polymer based assemblies.^5,8,10,11^ Polymeric systems are particularly versatile in this regard, benefiting from powerful synthetic tools, including controlled radical polymerisation to access vectors with tightly defined architectures and chemistries.^8,12,13^ In this context, polymer brushes, uniquely dense surface coatings, generated through controlled surface-initiated approaches, offer exceptional architectural control, including *via* their thickness, molecular weight and grafting density, parameters that in turn control their physicochemical properties.^13–18^ For gene delivery, polymer brushes grown from nanoparticle cores are especially attractive. Since polymer chains are covalently immobilised rather than freely assembled, they enable systematic variation of chemical structure while largely preserving overall architecture, independently of chemistry or core composition,^17–19^ with scope for post-polymerisation modification,^20^ collectively enabling rigorous characterisation of how chemical design affects RNA capture, release, and transfection efficiency. This degree of structural control is difficult to achieve with self-assembled lipid or polymer systems, yet is highly desirable for mechanistic studies in gene delivery, for instance, when investigating the interplay between RNA binding capacity and therapeutic outcome. In self-assembled systems, small changes in formulation can significantly impact the resulting architecture or structural integrity of resulting vectors and complexes, constraining the compositional landscape that can be probed. Therefore, this requires challenging synthetic and screening efforts to disentangle chemical from architectural factors.^21,22^

In particular, surface-initiated PDMAEMA (poly(2-dimethylaminoethyl methacrylate)) brushes enable stable siRNA adsorption through deep infiltration, resulting in high binding capacities,^23,24^ delivering sustained knockdown efficiencies over multiple days across a range of cell lines.^25–27^ Although cationic brushes appear to perform well for siRNA delivery, the impact of a broader range of brush polycation chemistries has not been investigated. Characterisation of these systems has relied predominantly on PDMAEMA and its quaternised derivatives; whether further optimisation of transfection efficiencies is possible, and whether other chemical and architectural properties, including hydrophobicity, p*K*_a_, and chain rigidity, play a significant role in RNA stabilisation and delivery efficiency, remains unclear. How structural differences in the side chains of grafted polycationic brushes influence key delivery parameters, including toxicity, binding capacity, and transfection kinetics, has not been systematically investigated. Upon cytosolic entry, polycationic vectors encounter concentrated cytoplasmic biomacromolecules and highly charged proteins that drive RNA release through competitive displacement.^27^ Notably, this process appears modulated by polycation binding strength, suggesting that polymer chemistry fundamentally governs temporal intracellular release and, consequently, therapeutic profiles.^27^

Here, we extend the library of tertiary and quaternary amine-bearing polymer brushes for RNA delivery, spanning a range of hydrophobicity (methyl to isopropyl substituents), p*K*_a_ (∼5 to 7), and chain rigidity (flexible alkyl *versus* rigid heterocyclic amine brushes). We systematically investigate how side chain modifications affect complexation and release kinetics of siRNA by surface plasmon resonance (SPR) and competitive binding assays, and investigate their broader impact on vector internalisation and gene silencing efficiency in human umbilical vein endothelial cells (HUVECs) by flow cytometry and immunofluorescence. We establish design rules connecting polymer chemistry, RNA binding and desorption properties, vector internalisation, and transfection kinetics for the rational optimisation of polycationic brush gene delivery vectors.

## Results and discussion

### Synthesis and chemical characterisation of polymer brushes

A series of alkyl and heterocyclic tertiary amine-bearing polymer brushes were synthesised to vary the hydrophobicity, p*K*_a_, and rigidity of polycationic brushes (Fig. 1A). Control over hydrophobicity was achieved by extending the length of alkyl chains on corresponding tertiary amines from methyl (PDMAEMA) to ethyl (poly(2-(diethylamino)ethyl methacrylate), PDEAEMA) and isopropyl (poly(2-(diisopropylamino)ethyl methacrylate), PDIPAEMA). In parallel, these polymers display a slight reduction in p*K*_a_ from 7.0 to 6.0,^28,29^ respectively. Heterocyclic polymers P2VP (poly(2-vinyl pyridine)) (p*K*_a_ ≲ 5.0 ^29,30^) and PVIM (poly(1-vinylimidazole)) (p*K*_a_ ∼6.0–6.9 ^31,32^) were selected to present more rigid side chains, due to their aromatic cyclic residues, compared to flexible alkyl methacrylates. All polymer chemistries except PVIM displayed consistent, approximately linear growth kinetics, as followed by ellipsometry, in particular at early time points (Fig. 1C–E).

**Fig. 1:**
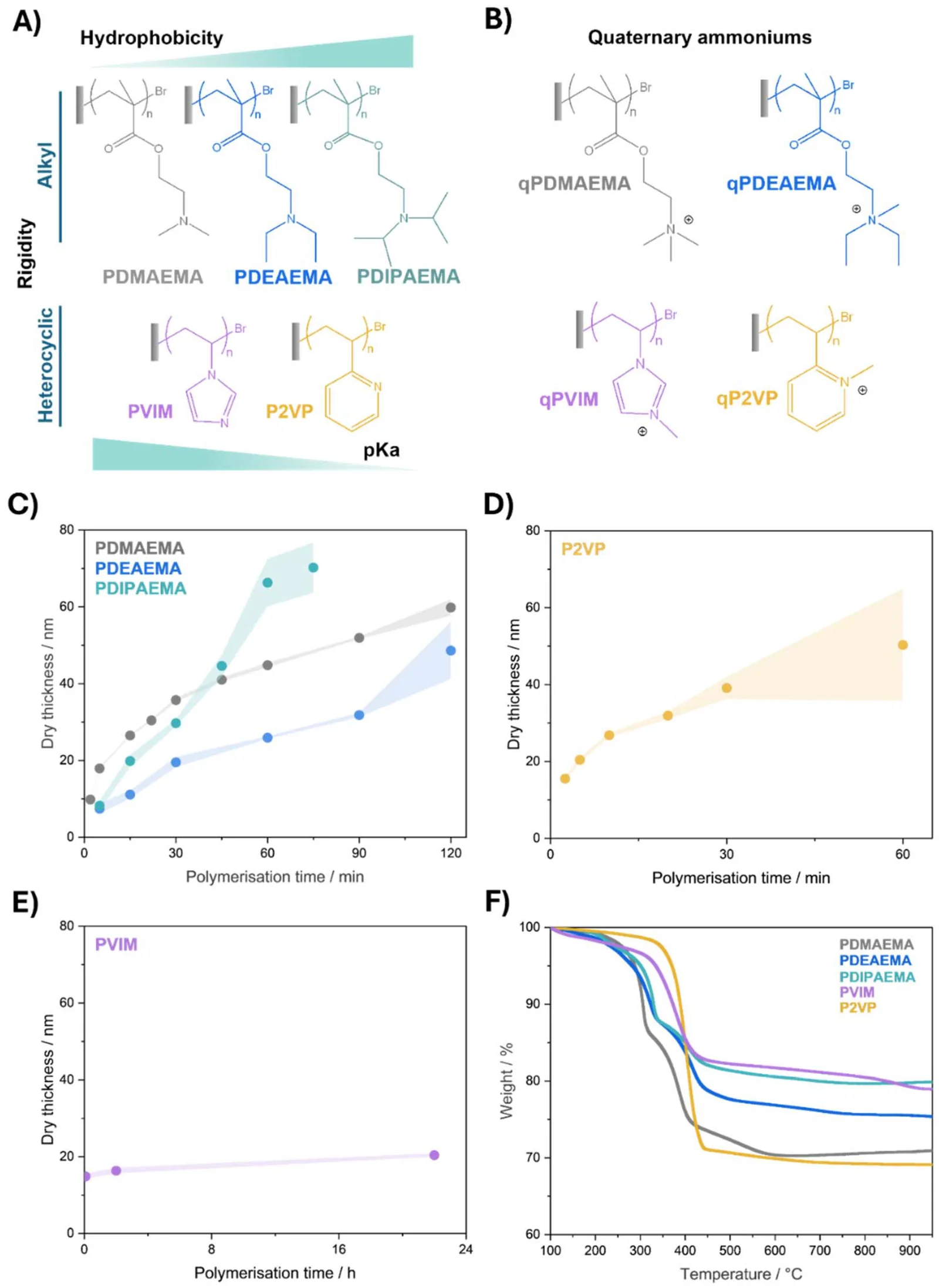
(A and B) Chemical structure of the cationic polymer brushes investigated in the present study. Gradient arrows denote hydrophobicity/p*K*_a_ changes within the series of polymers studied. (C–E) Evolution of ellipsometric thicknesses as a function of time for flexible (C; alkyl) and rigid (D and E; heterocyclic) cationic polymer brushes, generated *via* SI-ATRP. Shaded lines indicate error bars (s.e.m.; *N* ≥ 3). (F) TGA traces of corresponding polymer brushes generated from SiO_2_ nanoparticles (300 nm core; synthesised at polymerisation times targeting a brush thickness of 30 nm, or 20 nm for PVIM; see Table S1).

A target dry thickness (*h*_dry_) of 30 nm was achieved for all systems except for PVIM, which reached only 20 nm. The lack of control of PVIM brush growth is thought to be due to inherent challenges in the controlled polymerisation of *N*-vinyl monomers, with reduced resonance stabilisation of propagating radicals.^33^ Polymerisation kinetics were strongly influenced by solvent composition. For example, PDMAEMA brushes generated in 7 : 3 H_2_O : EtOH showed rapid yet controlled growth, mediated by the high solubility of the copper catalyst.^34^ More hydrophobic monomers required adjustment of the alcohol : water ratios to balance solubilisation and polymerisation control. MeOH and IPA were used as the majority solvent components for PDEAEMA and PDIPAEMA polymerisations, respectively, in agreement with the literature.^35^

For heterocyclic chemistries, brush synthesis required stronger ligands (Cyclam). In the case of P2VP, this was important to overcome competitive coordination of pyridine nitrogen with copper catalyst,^36^ producing ellipsometric growth kinetics comparable to PDMAEMA (Fig. 1C and D).

Similarly, polymer brushes were generated from SiO_2_ nanoparticles and characterised by TGA (Fig. 1F). Variations in mass loss were observed across the different brush chemistries. For polymethacrylate brushes, minor differences in decomposition steps originating from corresponding chemical structures were evident. A shift in thermal stability (methyl < ethyl < isopropyl) suggests that bulkier amine substituents modestly increase thermal stability (Fig. 1F). In contrast to alkyl amine methacrylate brushes, heterocyclic systems showed a single step decomposition, corresponding to aromatic rings. The decomposition onset was also delayed compared to alkyl derivatives, presumably due to the enhanced thermal stability expected for aromatic compounds. We also observed differences in the weight loss of P2VP and PVIM. Indeed, it has been reported that heterocyclic chemistries exhibit comparable thermal stability (reported for untethered polymers), with complete decomposition achieved by 500 °C.^37,38^ Therefore, higher weight loss from P2VP compared to PVIM may be associated with the difference in brush thickness (30 nm *vs*. 20 nm, respectively), assuming comparable grafting densities were generated. While the polymerisation kinetics of polymer brushes generated from curved surfaces can differ from those on planar surfaces,^39,40^ PDMAEMA has shown good correlation when generated from SiO_2_ nanoparticles *versus* flat silicon wafers, with comparable initiator monolayers.^26^ Therefore, at least for methacrylates, significant differences in brush growth across substrates are not expected. The TGA traces and thickness estimation for PDMAEMA were in good agreement with the literature.^26^

We further characterised the molecular weights of brushes cleaved from SiO_2_ nanoparticles and calculated corresponding grafting densities (Fig. S1). PDMAEMA and PDEAEMA brushes exhibited comparable molecular weights and high grafting densities (*M*_n_ = 62 and 59 kDa; *σ* = 0.47 and 0.39 chains per nm^2^, respectively). This is in good agreement with previous reports on dense, 30 nm-thick PDMAEMA brushes (*M*_n_ = 50 kDa; *σ* = 0.5 chains per nm^2^).^25^ Heterocyclic systems P2VP and PVIM were found to have slightly higher molecular weights (79 kDa and 65 kDa, respectively), with grafting densities of 0.40 and 0.29 chains per nm^2^. In contrast, PDIPAEMA brushes had chains with considerably larger molecular weights (*M*_n_ = 203 kDa) and lower grafting densities of 0.09 chains per nm^2^, already within the sparse regime.^26^

We next confirmed the chemistry of the brushes generated by GA-FTIR, with polymer brushes generated from flat silicon substrates coated with gold (for enhanced signal intensity). FTIR spectra (Fig. 2A and Fig. S2) of all polymethacrylate brushes showed a characteristic C=O stretching band associated with carbonyl bonds at 1730 cm^−1^. In agreement with changes in alkyl chain lengths, the intensity of C–H asymmetric stretching bands (2970 cm^−1^) gradually decreased in the following order: isopropyl > ethyl > methyl. Vibrational bands at 1390 cm^−1^ and 1465 cm^−1^ were also observed and were attributed to C–C bending modes.^41^ For all polymethacrylate brushes, relatively strong bands were also observed in the range of 1120–1200 cm^−1^. The most intense band at 1150 cm^−1^ was attributed to C–N stretching vibrations of the tertiary amines,^42,43^ while the weaker band at 1180 cm^−1^ was attributed to C–O stretching vibrations of the ester groups, typically reported in this range for poly(methacrylates).^44^ In the case of the heterocyclic amine brushes, vibrations from the pyridine and imidazole groups were evident at 1590/1570 cm^−1^ and 1500 cm^−1^, respectively. For PVIM, C–H ring stretching vibration was found at 3110 cm^−1^, while aromatic C–H stretch for P2VP was found at 3005 cm^−1^.^37,45^ For P2VP, vibrations at 1470 and 1435 cm^−1^ were attributed to the C=C stretch in the pyridine ring.

**Fig. 2:**
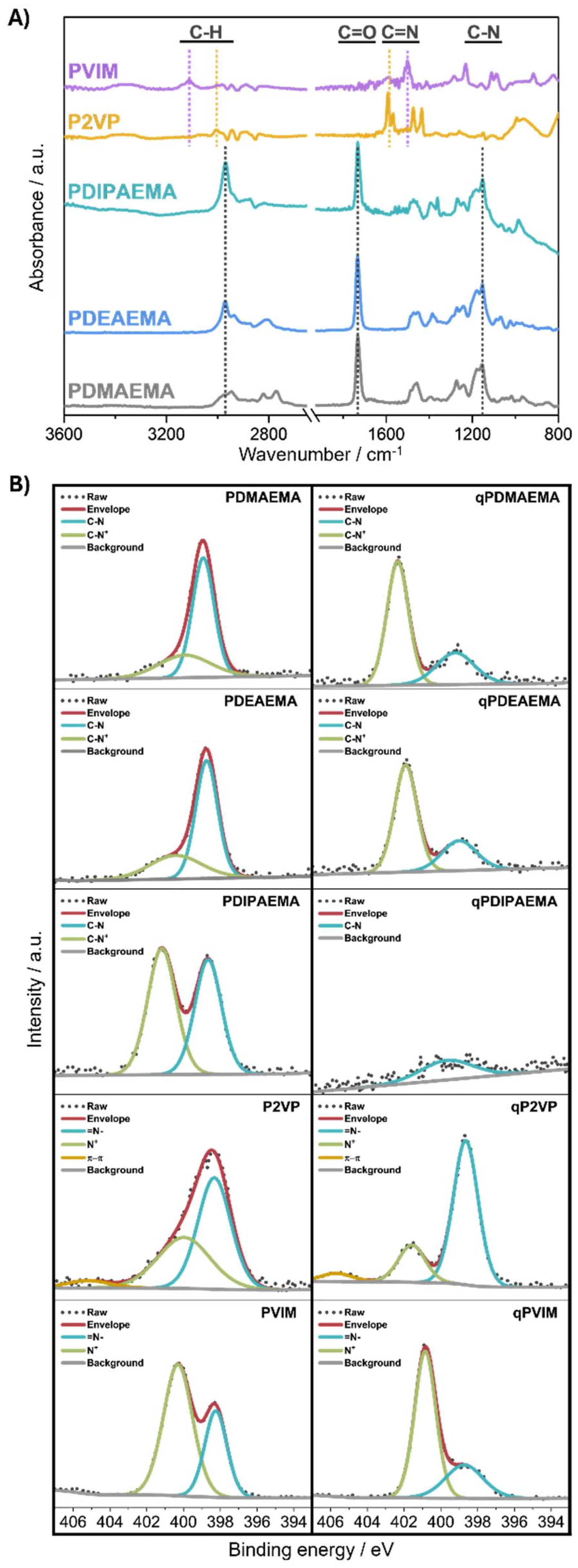
(A) FTIR spectra of tertiary amine-bearing polymer brushes generated from gold-coated silicon substrates. (B) High-resolution XPS analysis of N 1s in tertiary and quaternary amine-bearing polymer brushes generated from SiO_2_ nanoparticles (300 nm core).

XPS was used to further investigate the chemistry of the brushes generated from silica nanoparticles and flat silicon substrates (Fig. 2B and Fig. S3, S4). The N 1s peak at 400 eV confirmed the presence of tertiary amines in all brushes. For polymethacrylates, C 1s peaks corresponding to esters were found at 289 eV (Fig. S3 and S4).^27^ Similar to FTIR, high-resolution C 1s spectra of polymethacrylates evidenced an increased ratio of alkane (bands at 284–286 eV) to ester components (289 eV), with increased length of alkyl substituents. The high-resolution C 1s of P2VP showed a characteristic π–π* satellite at 292.5 eV associated with the delocalised electrons from the pyridine ring. This satellite was also observed in the N 1s spectrum (at 406.5 eV) next to the main peak associated with aromatic C (at 399 eV). Broader C 1s peaks for the imidazole ring in PVIM were observed, attributed to the higher contribution of adventitious carbon resulting from lower brush thickness. In contrast, more defined peaks were observed in the N 1s region at 400.6 and 399 eV, respectively. While the imidazole ring displays delocalisation of electrons, no satellite effect was observed, likely due to the reduced ring resonance associated with these moieties.

To isolate the effect of pH-responsiveness and brush hydrophobicity on RNA binding and release, we explored the quaternisation of all brush chemistries with methyl iodide. Quaternisation of tertiary amines was confirmed by high-resolution XPS spectra at C 1s and N 1s (Fig. 2B and Fig. S3). For alkyl methacrylate brushes, incorporation of an additional methyl group led to modest changes in the C 1s peak corresponding to alkyl amines, near 286 eV. A more pronounced increase in ammonium peaks (402 eV) was observed in N 1s spectra, with comparable extents of quaternisation observed for PDMAEMA and PDEAEMA brushes. However, for PDIPAEMA, we observed significant chain de-grafting accompanied by substantial hydrolysis (nitrogen contents reduced almost to background levels) (Fig. 2B). This is likely due to the significantly longer PDIPAEMA chains (*M*_n_ = 203 kDa), with bulkier side chains, which upon quaternisation experience strong electrostatic repulsion. When hydrated, these highly extended chains create mechanical stress that favours bond cleavage,^46^ leading to both degrafting and ester hydrolysis. Functionalisation of P2VP was less evident, as relatively weak changes in XPS spectra were observed. This is possibly due to the protonation of brush functionalised nanoparticles, which had to be dispersed in low pH to allow re-dispersion.

In agreement with XPS observations, we observed marked differences in the dry thickness of quaternised brushes (Fig. S5). The dry thickness of PDMAEMA brushes increased the most significantly (34%), in comparison to PDEAEMA (12%). The modest increase in thickness observed for PVIM and P2VP brushes (14%, and 5%, respectively) could reflect the weaker nucleophilic character of corresponding amines. This is in agreement with the more significant increase in the molar mass (and volume) of PDMAEMA repeat units upon methylation, compared to other methacrylate brushes. In agreement with XPS data, significant reduction in the thickness of PDIPAEMA was observed upon attempted quaternisation.

Therefore, overall our data indicate an excellent control of the production of 4 of the cationic polymer brushes targeted, with more mitigated success in production of PVIM brushes. Upon quaternisation, XPS data unambiguously confirmed the functionalisation of 4 of the brushes targeted, and attempted quaternised PDIPAEMA brushes were not considered further.

### Swelling behaviour by ellipsometry

We next studied the swelling and pH response of the cationic brushes generated, *via in situ* ellipsometry. For tertiary amine brushes, swelling was highly dependent on pH (Fig. 3A), in accordance with their expected p*K*_a_. PDMAEMA brushes exhibited highly extended chains with a swollen thickness of 80 nm (from 30 nm dry thicknesses) in PBS, at pH 7.4 (Fig. 3A). As the length of alkyl substituents increased, PDEAEMA and PDIPAEMA displayed progressively reduced swelling in identical conditions. PDEAEMA swelling was only achieved at pH of 5.5, likely reflecting the combined increase in hydrophobicity and modest reduction in p*K*_a_. Compared to untethered polymers, grafted polymers exhibit high interchain repulsion that significantly impact their solution conformation.^47,48^ Therefore, in addition to changes in steric hindrance near the amine moieties and hydrophilicity of substituents, shifts in the p*K*_a_ of polycationic brushes as a function of grafting density,^47^ ionic strength^35^ are not surprising.

**Fig. 3:**
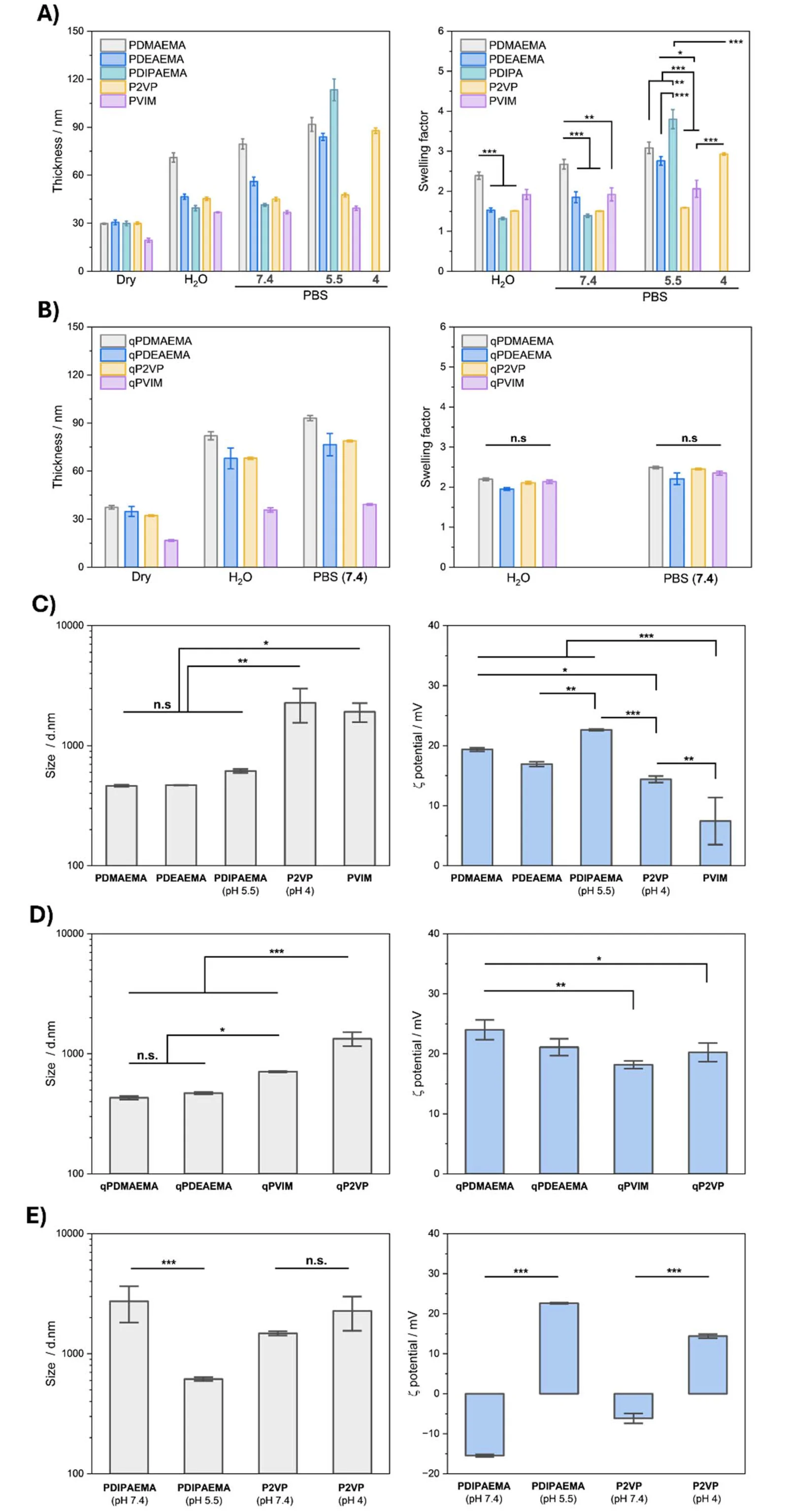
Swelling behaviour and colloidal stability studies of surface-initiated polymer brushes by ellipsometry and DLS, respectively. (A and B) Swollen thickness (left) and swelling factors (right) of tertiary amines and quaternary ammoniums (generated from flat substrates) in H_2_O and PBS (at different pH), at RT. We note that quaternisation fully reversed the hydrophobicity of P2VP at pH 7.4 (B). (C–E) Hydrodynamic diameters (left) and *ζ* potentials (right) of polymer brush-functionalised SiO_2_ nanoparticles (300 nm core) in PBS at 25 °C (evaluated in PBS pH 7.4, unless otherwise stated), measured for cationic polymer brushes before (C) and after (D) quaternisation. (E) Impact of pH on hydrodynamic diameters and *ζ* potentials of selected tertiary amine brushes. Error bars represent s.e.m (for A and B) and s.d (for C–E); *N* = 3. *p < 0.05; **p < 0.01; ***p < 0.001.

To characterise the fully extended swelling of polymethacrylate brushes, incubation in PBS with a pH of 5.5 was studied. Surface-initiated PDEAEMA and PDIPAEMA brushes were previously shown to swell below pH 7.5 and 6.5, respectively (incubated in 10 mM phosphate buffer solutions).^35^ While comparable swelling behaviour was found for PDMAEMA and PDEAEMA, their isopropyl counterpart, PDIPAEMA, exhibited a significantly higher swelling factor (Fig. 3A). This likely reflects the lower grafting density achieved with these brushes (Fig. S1) and inherent interchain repulsion from the sterically hindered isopropyl groups.

For heterocyclic cationic brushes, P2VP displayed significant swelling only at very low pH (pH 4), in agreement with its low p*K*_a_. However, when fully protonated, it reached comparable swelling to that of PDMAEMA. In contrast, PVIM showed rather limited swelling across the different buffer systems, with modest swelling factors compared to other brushes investigated. Since PVIM has been reported to be protonated at slightly acidic pH, including pH 5.5 (p*K*_a_ ∼ 6.0–6.9 ^31,32^), limited swelling even at this pH suggests a lower protonation than anticipated from its solution p*K*_a_. The striking similarity between the different swelling factors observed upon quaternisation implies comparable conformation flexibility, despite the rigid aromatic moieties introduced in P2VP and PVIM brushes.

### Colloidal stability of brush-functionalised nanoparticles

Following the characterisation of the solution behaviour of cationic brushes tethered to planar substrates, the response of brushes grafted from SiO_2_ nanoparticles was investigated *via* dynamic light scattering (DLS), in PBS (150 mM) (Fig. 3C–E). PDIPAEMA- and P2VP-functionalised nanoparticles were incubated at pH 5.5 and 4, respectively, to ensure maximum swelling and avoid aggregation. In agreement with the ellipsometric swelling observed, comparable particle hydrodynamic diameters were measured for the three fully protonated polymethacrylate brushes, with *ζ*-potentials near 20 mV. In addition, we confirmed the pH-responsiveness of PDIPAEMA and P2VP, with a clear reversal of the *ζ*-potential at acidic pH (from negative to positive values) (Fig. 3E). For PDIPAEMA, this yielded hydrodynamic diameters comparable to those of PDMAEMA. However, this was not the case for P2VP, which formed relatively large aggregates ≥1.5 µm (even at pH 4), associated with comparatively low *ζ*-potentials (*e.g.*, compared to PDMAEMA). We attributed this to a combination of the inherently high hydrophobicity and incomplete protonation of P2VP, which would require incubation at lower pH for full protonation, but may remain poorly solvated owing to the hydrophobicity of pyridine residues. Similarly, PVIM, despite a higher hydrophilicity, formed large aggregates (∼1.9 µm) and also showed poor colloidal stability (*ζ*-potential of 7 mV). We note, however, that this polymer was only incubated at pH 7.4 and therefore was likely not sufficiently protonated.

However, quaternisation of PVIM greatly improved its colloidal stability (Fig. 3D), since large aggregates were not observed, and indeed, diameters in qPVIM were only slightly higher compared to PDMAEMA. Conversely, the limited quaternisation achieved for P2VP did not overcome its hydrophobicity and hydrodynamic diameters remained relatively unchanged (compared to diameters at pH 7.4). However, a slight increase in *ζ*-potential was observed. Quaternised PDMAEMA and PDEAEMA brush-decorated nanoparticles behaved similarly to their tertiary amine counterparts, with comparable hydrodynamic diameters and slightly increased *ζ*-potentials. The colloidal properties of brush-functionalised nanoparticles are particularly important for gene delivery since poor stability and particle aggregation reduce cellular internalisation, and impact cytotoxicity.^17^ Therefore, this evaluation allowed us to confirm the suitability of the brushes investigated for further transfection assays.

In addition, we determined the *ζ*-potential of brush-nanoparticles in diluted PBS (0.1×) (SI Fig. S6) to reduce the effect of high ionic strength in buffer, which is known to screen charges and compress the electrical double layer, reducing the actual surface charge density.^49^ Overall, characterisation of surface charges in low ionic strength buffer was in good agreement with the trends observed when nanoparticles were dispersed in full PBS. However, differences across chemistries observed initially in full PBS, were now more pronounced. For polymethacrylates brushes, *ζ*-potentials decreased with decreasing p*K*_a_, as follows: 24 mV, 8 mV and −20 mV, for methyl, ethyl and isopropyl amine substituents, respectively. P2VP and PVIM displayed values of −5 mV and −12 mV, respectively. Finally, we observed larger increases in surface charge upon quaternisation, consistent with a weakened surface charge density at higher ionic strength (Fig. S6B).

### RNA binding cationic polymer brushes

The ability of polymer brushes to bind siRNA (20 bp) was investigated by SPR in PBS at different pH, to ensure the complexation conditions (pH) enabled RNA capture (Fig. 4). Based on ellipsometry and DLS studies, the pH of the buffer was adjusted, depending on the targeted brush, to enable protonation (*e.g.*, pH 5.5 or 4 for polymethacrylates and PVIM and P2VP, respectively). In agreement with their pH-responsiveness, we found the complexation of RNA to be largely dependent on protonation levels and swelling. At pH 7.4, PDMAEMA complexed siRNA at a maximum surface density of 3.9 µg cm^−2^, followed by PDEAEMA and PDIPAEMA at 2.4 µg cm^−2^ and 0.04 µg cm^−2^, respectively (Fig. 4A). However, when these brushes were in environments below their p*K*_a_, binding capacities were comparable. This is an important design insight as it indicates that the increased interchain repulsion experienced by flexible brushes with bulkier hydrophobic alkyl substituents does not restrict RNA infiltration and effective packing. Moreover, polymers displaying a low p*K*_a_ (*e.g.*, PDIPAEMA) clearly complexed RNA less stably near neutral pH, leading to premature payload release upon exposure to transfection media (pH 7.4) and consequently poor transfection efficiency.

**Fig. 4:**
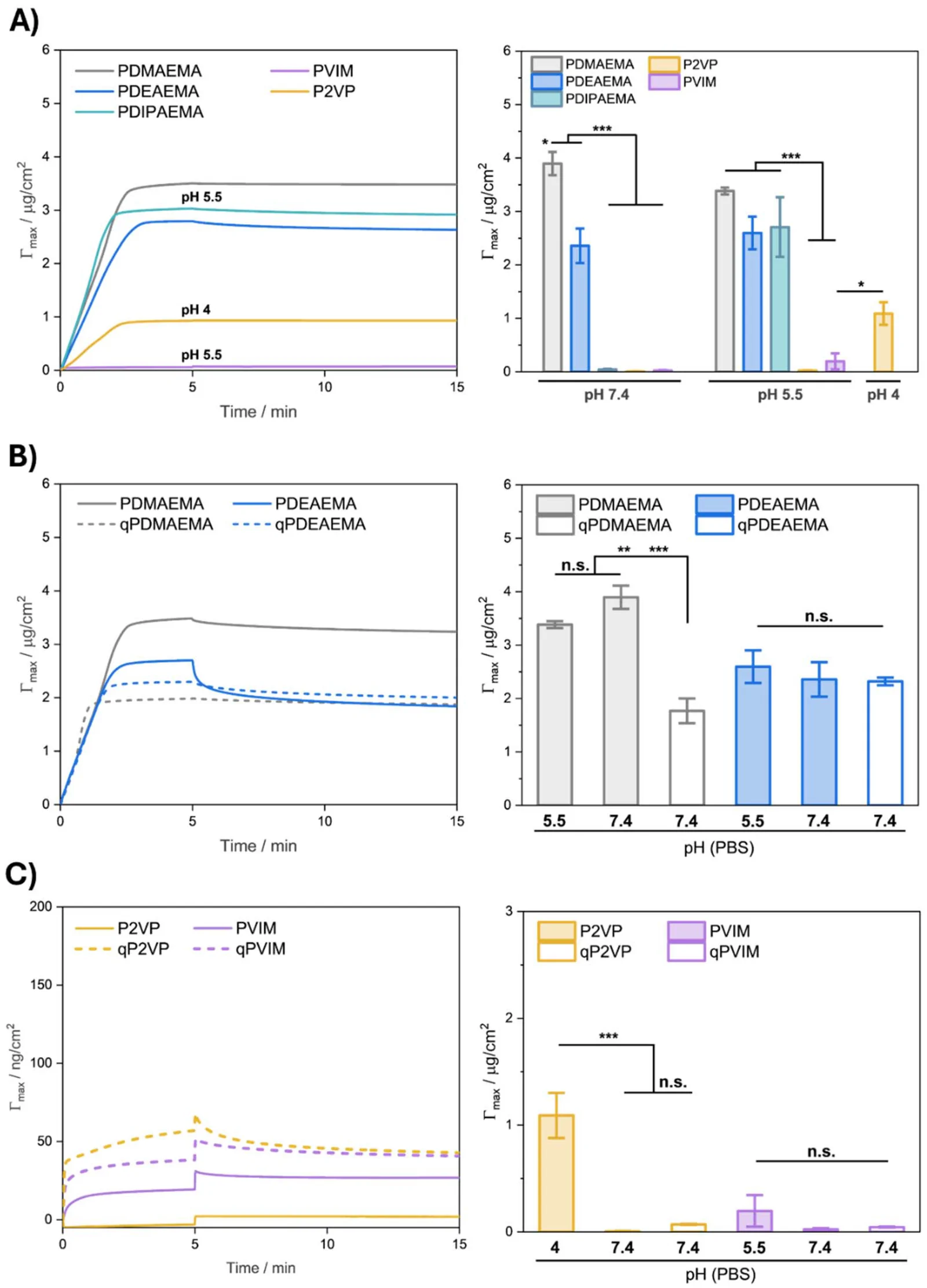
Adsorption of siRNA (20 bp) to (A) tertiary amine-polymer brushes and (B and C) quaternised polymer brushes compared to tertiary counterparts, characterised by SPR in PBS, at 25 °C. Left: Representative SPR traces at selected pH to ensure brush protonation (in A) and at pH 7.4 in (B and C). Ultimate surface densities extracted from SPR traces (right). Error bars represent s.e.m.; *N* ≥ 3. *p < 0.05; **p < 0.01; ***p < 0.001.

Notably, heterocyclic amines showed drastically reduced RNA binding capacity compared to flexible polymethacrylate brushes. For P2VP (at pH 4) and PVIM (at pH 5.5), siRNA surface densities measured were 1.1 µg cm^−2^ and 0.2 µg cm^−2^, respectively (Fig. 4A). This is perhaps reflecting the changes in flexibility and hydrophobicity associated with the brushes, but more importantly these observations likely reflect the reduced surface charge measured for tertiary amine brushes.

The binding of siRNA to quaternised polymer brushes was next evaluated (Fig. 4B and C). We first noted marked differences when comparing qPDMAEMA and qPDEAEMA to their non-quaternised counterparts (Fig. 4B). At both pH 7.4 and pH 5.5, qPDMAEMA displayed lower binding of siRNA (1.8 µg cm^−2^) compared to PDMAEMA. This was attributed to loss of hydrogen bonding capacity, proposed to contribute to interactions with RNA and stabilisation within the brush (unprotonated amines can interact with hydroxyls in the 2′ position of the ribose backbone).^24^ In contrast, we observed no difference in RNA adsorption to qPDEAEMA (2.3 µg cm^−2^) compared to PDEAEMA, even at pH 5.5 when PDEAEMA is highly swollen. This suggests that, unlike binding to PDMAEMA, PDEAEMA capture of siRNA oligos is primarily dominated by electrostatic interactions, perhaps due to the increased steric hindrance preventing hydrogen bonding. As a result, adsorption of siRNA oligos was comparable in qPDEAEMA, compared to qPDMAEMA. Since the only structural difference between these two polymers is the increased hydrophobicity from ethyl side chains, we conclude that such modest change in hydrophobicity and associated swelling has little impact on the comparatively long-range electrostatic interactions of corresponding quaternary ammonium centres with phosphates.

Finally, we studied the impact of quaternisation of rigid polymer brushes on siRNA binding (Fig. 4C). In qPVIM and qP2VP, adsorption levels remained low compared to poly(methacrylates) studied, particularly for qPVIM (0.05 µg cm^−2^). However, qP2VP displayed a modest increase in RNA binding at pH 7.4 compared to non-quaternised P2VP at the same pH (0.07 µg cm^−2^ for qPV2VP). This remained considerably lower than the binding observed for P2VP at pH 4. Therefore, despite comparable swelling, the infiltration of 20 bp RNA oligos within rigid polymer brushes (qPVIM and qP2VP) was negligible and macromolecules likely remained adsorbed at the surface of these brushes. This could also reflect the reduced level of quaternisation observed for these brushes and their weak electrostatic surface potential. Overall, our observations suggest a strong impact of brush flexibility, swelling and electrostatic potential on RNA uptake and deep infiltration.

### RNA competitive desorption is modulated by the brush chemistry

Upon internalisation of vectors, competitive binding within the crowded cytosolic macromolecular environment regulates the dissociation of RNA–polymer complexes.^27^ Understanding these processes is particularly important to control and tune the processes *via* which RNA is released upon cytosolic entry and to promote short-term to prolonged transfection efficiencies. To gain further insights into the dissociation of brush-RNA complexes, we studied the desorption of RNA in the presence of whole-cell lysates by competitive binding assays (Fig. 5). In good agreement with previous reports,^27^ we observed the rapid dissociation of RNA from the surface of polymer brush-coated microparticles (3 µm core) upon exposure to cytosolic lysates from endothelial cells. As was previously observed in the case of exchange with fibroblast lysates, slower desorption from qPDMAEMA brushes was observed, compared to PDMAEMA (Fig. 5A). In contrast, desorption from qPDEAEMA was identical to that from PDMAEMA, and therefore faster than desorption from qPDMAEMA brushes. This indicates that even minor shifts in hydrophobicity impacts electrostatic interactions, resulting in faster RNA competitive desorption.

**Fig. 5:**
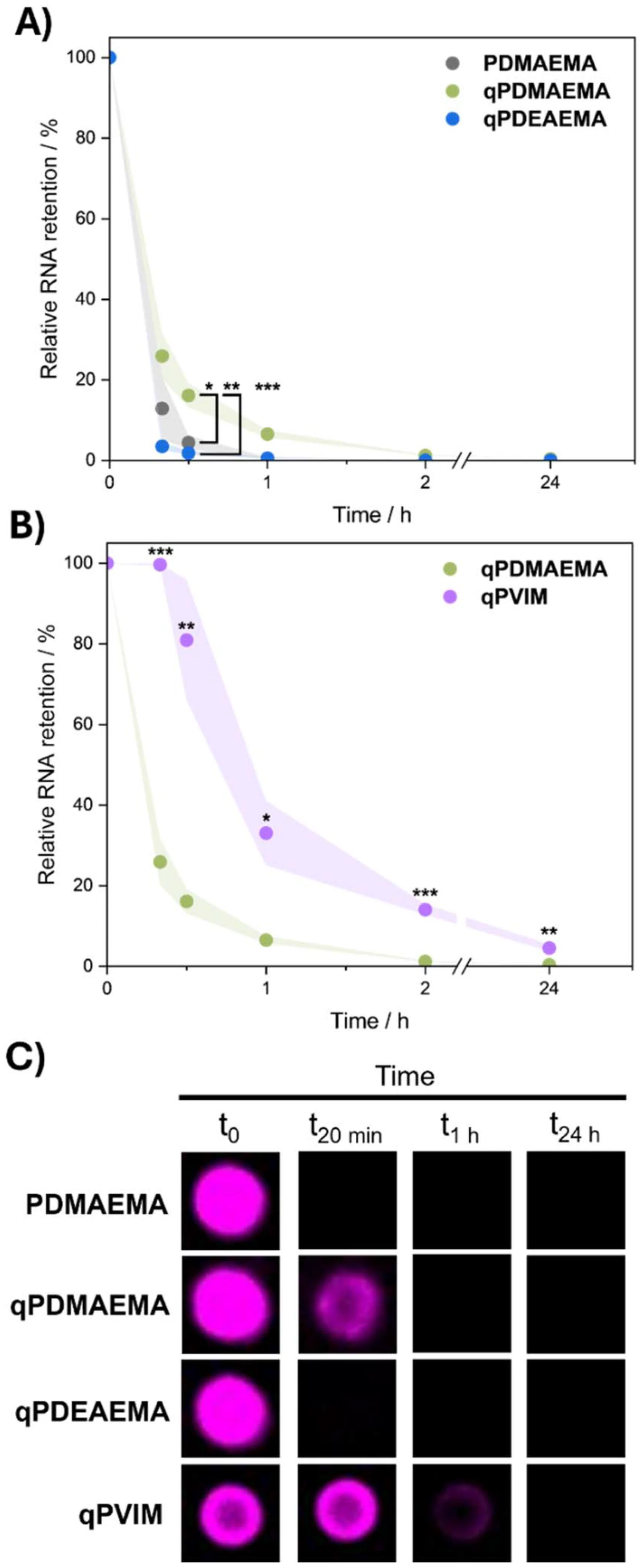
Competitive desorption of Cy5-tagged siRNA from polymer brush-functionalised SiO_2_ microparticles (3 µm core) in the presence of whole-cell endothelial lysate at 37 °C. (A and B) Quantification of the reduction in fluorescence intensity within 24 h. (C) Representative fluorescence micrographs. Error bars represent s.e.m.; *N* = 3. *p < 0.05; **p < 0.01; ***p < 0.001.

We previously reported that overall affinity constants of siRNA adsorption to PDMAEMA and qPDMAEMA are comparable.^24^ In both brushes, siRNA oligos were found to be deeply infiltrated within the brush structures.^23,24^ These observations are consistent with a two-stage process regulating the adsorption of RNA to polymer brushes. First, RNA rapidly adsorbs at the brush surface due to strong electrostatic interactions with cationic moieties, followed by subsequent infiltration throughout the brush, a process particularly sensitive to brush chain conformational rearrangement and oligonucleotide relaxation, in addition to the displacement of bound hydration layers. The increased hydrophobicity of qPDEAEMA, with weakened hydration layer, facilitates water displacement by RNA molecules during infiltration, resulting in higher RNA adsorption compared to qPDMAEMA. However, this leads to faster displacement, potentially due to the weaker electrostatic forces restricting RNA mobility. Therefore, we propose that brush hydration and the strength of electrostatic interactions play a critical role in the complexation and competitive desorption of RNA from polymer brush complexes.

In contrast, although we observed weak infiltration and loading of RNA oligos within qPVIM by SPR (which was confirmed by siRNA fluorescence intensity levels twofold lower compared to PDMAEMA), we found that their desorption was significantly delayed compared to qPDMAEMA (Fig. 5B). This suggests that whilst chain rigidity restricts molecular diffusion within qPVIM brushes and limits deep infiltration, the surface adsorbed RNA may be more tightly bound. This may reflect a higher binding affinity measured for these brushes, with increased rates of adsorption and reduced rate of desorption (despite a considerably reduced overall binding capacity and surface density, Fig. 4C). This is reminiscent of the properties of sparse PDMAEMA brushes, displaying high binding affinity, yet low binding capacity compared to their denser counterparts.^26^

### Transfection and knockdown (KD) efficiency with polycationic brushes

To assess the ability of the generated brush chemistries to deliver siRNA and knock down specific gene expression, we quantified the reduction of expression of the cell membrane protein platelet endothelial cell adhesion molecule 1 (PECAM-1), associated with leukocyte and immune cell adhesion and trans-endothelial migration.^50^ We first characterised the relative protein expression of PECAM-1 by immunofluorescence. At days 1 and 3 (Fig. 6 and SI Fig. S8, S9), we found that the KD efficiency largely depended on the p*K*_a_ of the corresponding brush. Only brushes with p*K*_a_ values near neutrality, PDMAEMA and PDEAEMA, induced significant KD, reducing protein expression to 39% and 55% at day 1, and further to 11% and 24% at day 3, respectively. In both cases, KD values were comparable to those of the positive control Lipofectamine (LP3000). Nevertheless, we noted significant cytotoxicity from PDEAEMA brushes, with only ∼50% cell viability and a significant reduction in cell density (SI Fig. S10). This is likely associated with more hydrophobic chains in PDEAEMA, which may adversely interact with membrane phospholipids and induce permeabilisation.^51^ Interestingly, we did not observe further increase in toxicity with PDIPAEMA, which rather showed good cell viability. Owing to its greater hydrophobicity, PDIPAEMA has a tendency to backfold toward the carbonyl groups/backbone to minimise water contact,^52^ potentially limiting disruptive interactions with cellular membranes. Its lack of cytotoxicity under the culture conditions tested could also stem from its reduced surface charge density, compared to PDEAEMA and PDMAEMA. This indicates that careful balance of hydrophobicity dictates the cytotoxicity of polycationic brushes, as is well established in the context of antibacterial properties.^16^ In agreement with this analysis, reducing the hydrophobicity of PDEAEMA (*via* quaternisation) effectively lowered cytotoxicity (SI Fig. S10).

**Fig. 6:**
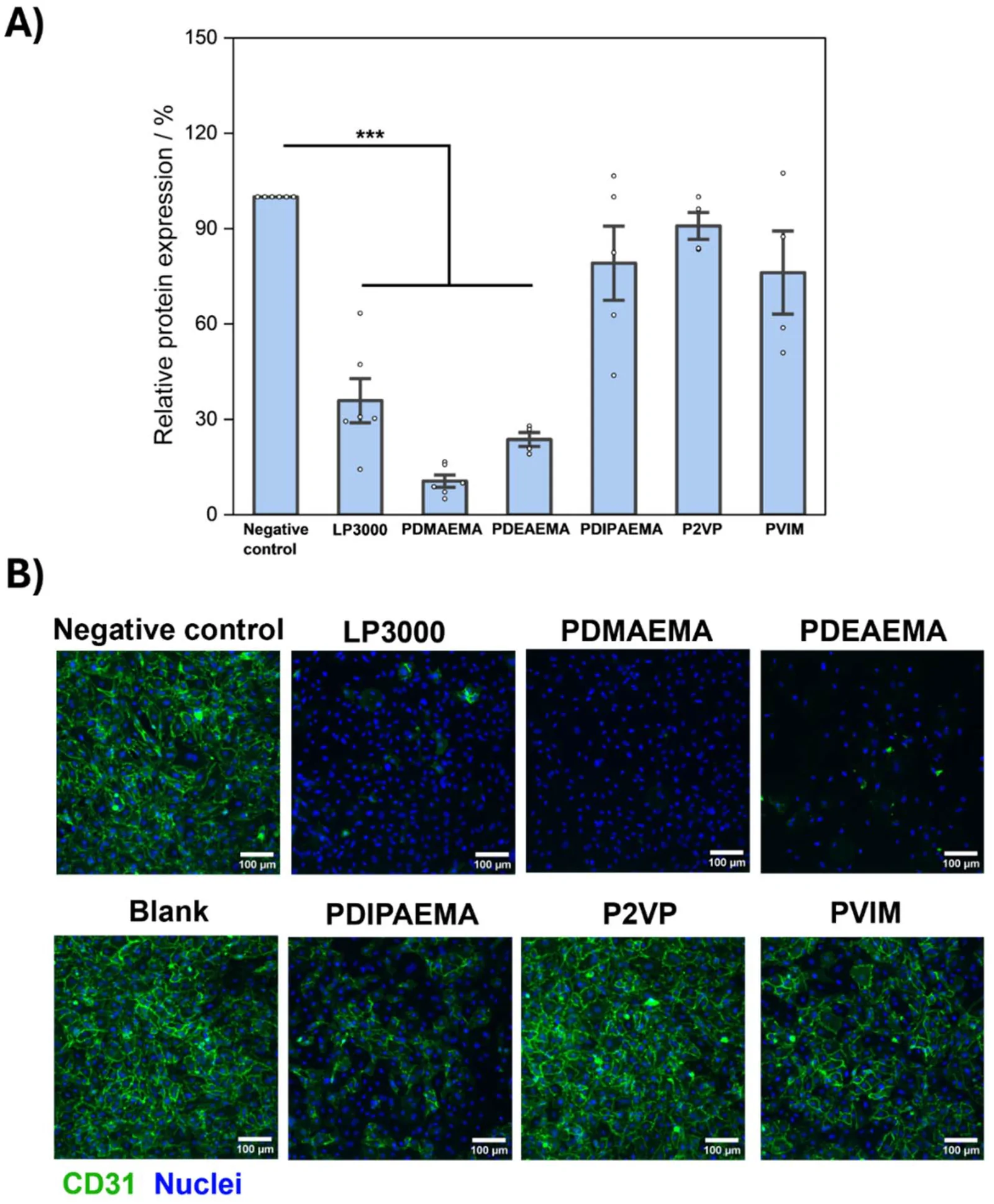
PECAM-1 knockdown efficiency in HUVECs at day 3 post-transfection with polymer brush-functionalised SiO_2_ nanoparticles (300 nm core). (A) Quantification of relative protein expression normalised to LP3000 negative control. (B) Representative immunofluorescence images showing PECAM-1 expression (green) and nuclear staining (blue). Error bars represent s.e.m.; *N* ≥ 4. *p < 0.05; **p < 0.01; ***p < 0.001.

Finally, for chemistries exhibiting p*K*_a_ below that of PDMAEMA (*i.e.* PDIPAEMA, P2VP, and PVIM), we did not observe any significant KD (Fig. 6 and SI Fig. S8, S9). While we initially incubated PDIPAEMA and P2VP at the pH of maximum adsorption (5.5 and 4.0, respectively), upon contact with transfection media (pH 7.4), these polycations deprotonate and release the RNA payload, retaining only low RNA densities (SI Fig. S7). Deprotonation of low p*K*_a_ chemistries is also reflected in their effective surface charge, displaying negative to near neutral *ζ* potentials at pH 7.4 (Fig. 3E). Therefore, these chemistries not only have weaker potential for RNA capture but are also less likely to be internalised, preventing effective delivery of RNA.

In contrast to low p*K*_a_ tertiary amines exhibiting no apparent KD, we found that all quaternised chemistries triggered significant KD of PECAM-1, to levels comparable to PDMAEMA and LP3000 (SI Fig. S8 and S9). Cytotoxicity among all quaternised brushes was comparable, including in the case of qPDEAEMA (SI Fig. S10). Therefore the hydrophobicity of ionisable brushes seems to be underpinning cytotoxicity in polymer brush delivery vectors and modestly hydrophobic quaternised brushes display comparatively low cytotoxicity, in particular compared to Lipofectamine, well-suited for transfections.

Following our initial screening, we selected the chemistries that achieved significant KD at early time points, *i.e.*, PDMAEMA, qPDMAEMA, qPDEAEMA, and qPVIM, and extended the study to assess transfection efficiency up to 14 days post-transfection (Fig. 7 and Fig. S11, S12). qP2VP is not presented in the extended study due to significant aggregation observed by DLS; the implications of this colloidal instability for cellular behavior and transfection efficiency are discussed later in this report. qPDEAEMA exhibited identical KD profiles to PDMAEMA, with high KD at day 3 and rapid recovery at day 7, highlighting its strong similarity in competitive desorption kinetics to PDMAEMA, despite qPDEAEMA binding slightly less RNA by SPR. The KD profiles of qPDMAEMA also correlated with our competitive binding assays, as we observed a significant delay in the KD at day 1, yet less pronounced KD levels at day 3 compared to PDMAEMA and qPDEAEMA. Protein expression levels gradually returned to normal control levels on subsequent days. This is in partial contrast with previous reports where qPDMAEMA outperformed PDMAEMA at intermediate-to-late time points in HaCaT cells, although this was targeting the expression of GFP-tagged actin engineered in this cell line.^27^ However, this is in good agreement with the competitive binding profiles, which showed slower release in the case of qPDMAEMA, using HaCaT cell lysates. Therefore, cytosolic biomacromolecular content and associated competitive binding correlate with the long term expression profile following silencing.

**Fig. 7:**
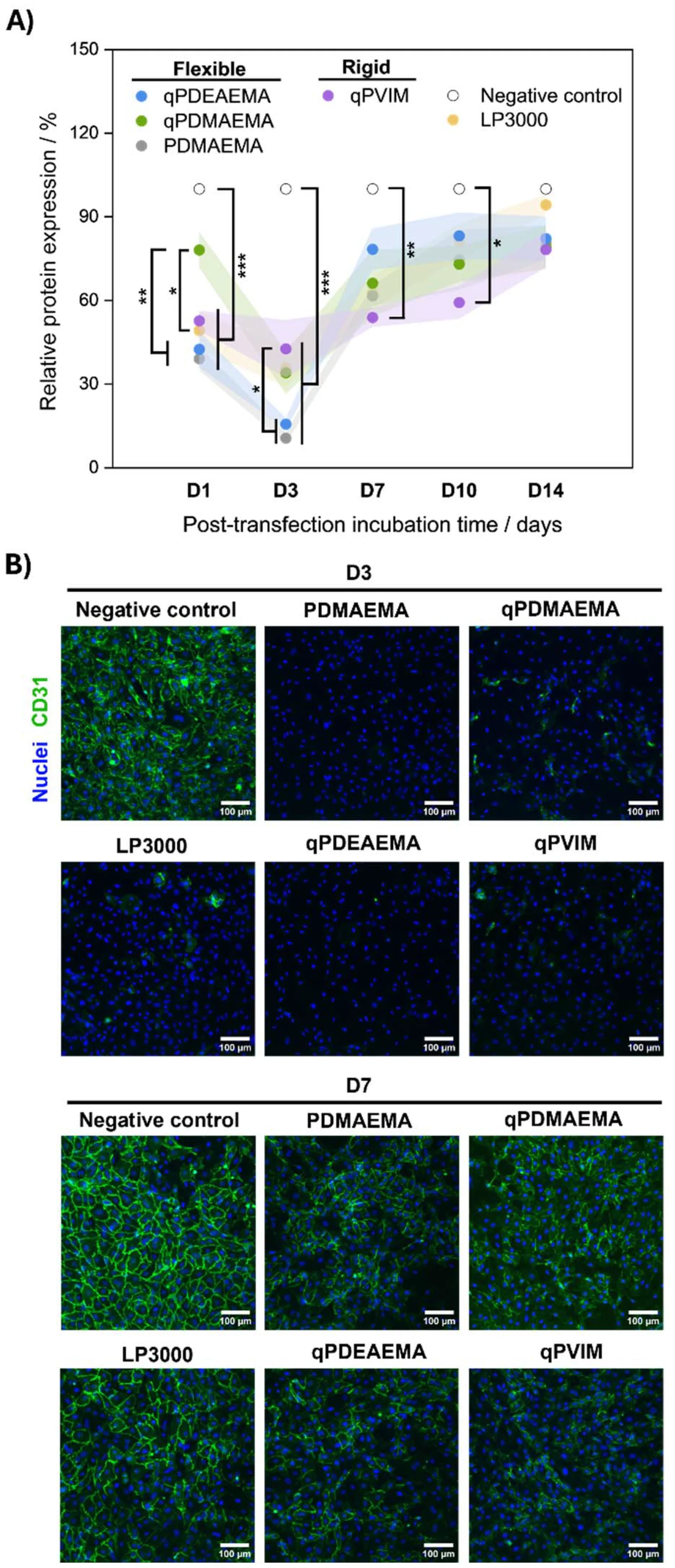
Transfection of kinetics of PECAM-1 knockdown efficiency in HUVECs with polymer brush-functionalised SiO_2_ nanoparticles (300 nm core). (A) Quantification of relative protein expression over 14 days post-transfection, normalised to LP3000 negative control. Error bars represent s.e.m.; *N* = 6 for D1–D10; *N* = 4 for D14. *p < 0.05; **p < 0.01; ***p < 0.001. (B) Representative immunofluorescence images at day 3 (maximum knockdown) and day 7 post-transfection. PECAM-1 expression (green) and nuclei (blue); scale bars: 100 µm. Details of quantified expression levels and corresponding images for D7–14 time points are shown in Fig. S11 and S12.

Remarkably, qPVIM achieved effective KD comparable to PDMAEMA at 1 day post-transfection, at the same N/P ratio, and despite complexing far less RNA (Fig. 4). qPVIM exhibited a unique temporal profile characterised by less profound initial silencing compared to PDMAEMA and qPDEAEMA brushes (see KD at day 3) but more sustained reduction in expression of the targeted protein over time (at ≥7 days). Specifically, PECAM-1 expression levels after qPVIM-mediated transfection remained at 54% and 59% at days 7 and 10, respectively, whereas PDMAEMA and LP3000 led to protein expression recovery, at the same time points (62% and 75%, and 63% and 81%, respectively). It is not clear how qPVIM enables such prolonged KD, despite the weaker apparent RNA uptake and binding affinity. This suggests an enhanced sequestration and protection within the brush, reflected in its competitive displacement profile (Fig. 5), allowing long term release of intact RNA. Although RNA absorption levels were found to be lower in the case of qPVIM, compared to PDMAEMA, qPDMAEMA and qPDEAEMA (Fig. 4), we cannot exclude an impact of the curvature of nanoparticles on the swelling and conformation of qPVIM brushes, potentially impacting RNA capture from corresponding nano- and micro-particles. It could also indicate different fate of the nanoparticles upon cytosolic entry.

Finally, while qP2VP achieved effective KD levels at early time points, comparable to those achieved with PDMAEMA (Fig. S8 and S9), this chemistry exhibited unusual behaviour at later time points that complicated interpretation of its transfection efficiency (Fig. S13). Indeed, at later time points, PECAM-1 expression levels remained particularly low, with KD efficiencies of 30% and 28%, at days 7 and 10, respectively. However, at these time points, both targeting and non-targeting siRNA produced similar reductions in PECAM-1 expression. Caspase-3 staining ruled out apoptotic cell death as a primary contributor to this behaviour (Fig. S13B). These observations could reflect the poor colloidal stability of P2VP-decorated nanoparticles, associated with their increased hydrophobicity. This could interfere with membrane organisation and/or antibody accessibility, since it is known that particles can access and accumulate at cell junctions rather than being internalised.^53^ Therefore, qP2VP was excluded from our conclusions on long-term studies as a potentially unsuitable candidate chemistry and displaying abnormal impact on protein expression more generally, rather than specific KD of a target protein.

### Internalisation of RNA mediated by cationic polymer brush-nanoparticles

To investigate how effective RNA payload concentrations influence transfection, we characterised cellular internalisation by flow cytometry and microscopy, using Cy5-tagged siRNA, 24 h post-transfection (Fig. 8 and Fig. S14). Flow cytometry data revealed distinct internalisation patterns, depending on polymer brush chemistry (Fig. 8A), and varied internalisation efficiencies (shown as median fluorescence intensity, MFI; Fig. 8C and as Cy5-positive cells; Fig. S14). It should be noted that all samples were prepared after incubation of cells with heparin, a rapid and quantitative competitor displacing RNA from brushes. Hence fluorescence intensity levels measured *via* flow cytometry and microscopy are not due to RNA/nanoparticles adsorbed at the outer surface of cell membranes. Among pH-responsive tertiary amine brushes, PDMAEMA demonstrated substantially higher RNA internalisation than all other conditions, as evidenced by both an increased proportion of Cy5-positive cells (97%; Fig. S14), and peak height in histogram (Fig. 8A) with elevated fluorescence intensity per cell (shift of the profiles towards higher intensities). This superior internalisation is consistent with the superior RNA binding capacity of PDMAEMA at physiological pH demonstrated by SPR measurements (Fig. 4). In contrast, low p*K*_a_ chemistries (PDIPAEMA, P2VP, PVIM) resulted in significantly less RNA internalisation, in good agreement with their reduced binding (by SPR) and unfavourable surface charge at physiological pH. PDEAEMA also showed reduced internalisation, which was attributed to its cytotoxicity. Notably, LP3000 led to only 7% (MFI) of the RNA delivered by PDMAEMA (Fig. 8C), despite achieving comparable level of RNA internalisation (Cy5-positive cells; Fig. S14) and comparable KD levels, and exhibited a particularly broad peak spanning several orders of magnitude in intensities, reflecting highly heterogeneous cell-to-cell delivery. We stress that the same dose of RNA was introduced in the transfection assays for all conditions. This indicates that internalised RNA levels are not the sole determinants of silencing efficiencies, and that subsequent processes, such as endosomal escape and RNA release from the vectors upon cytosolic entry, may significantly differ depending on the vector and brush chemistry. In addition, an RNA threshold for silencing, followed by dose-dependent saturation possibly modulated by RISC loading kinetics may also contribute to the modulation of KD efficiencies.^54^

**Fig. 8:**
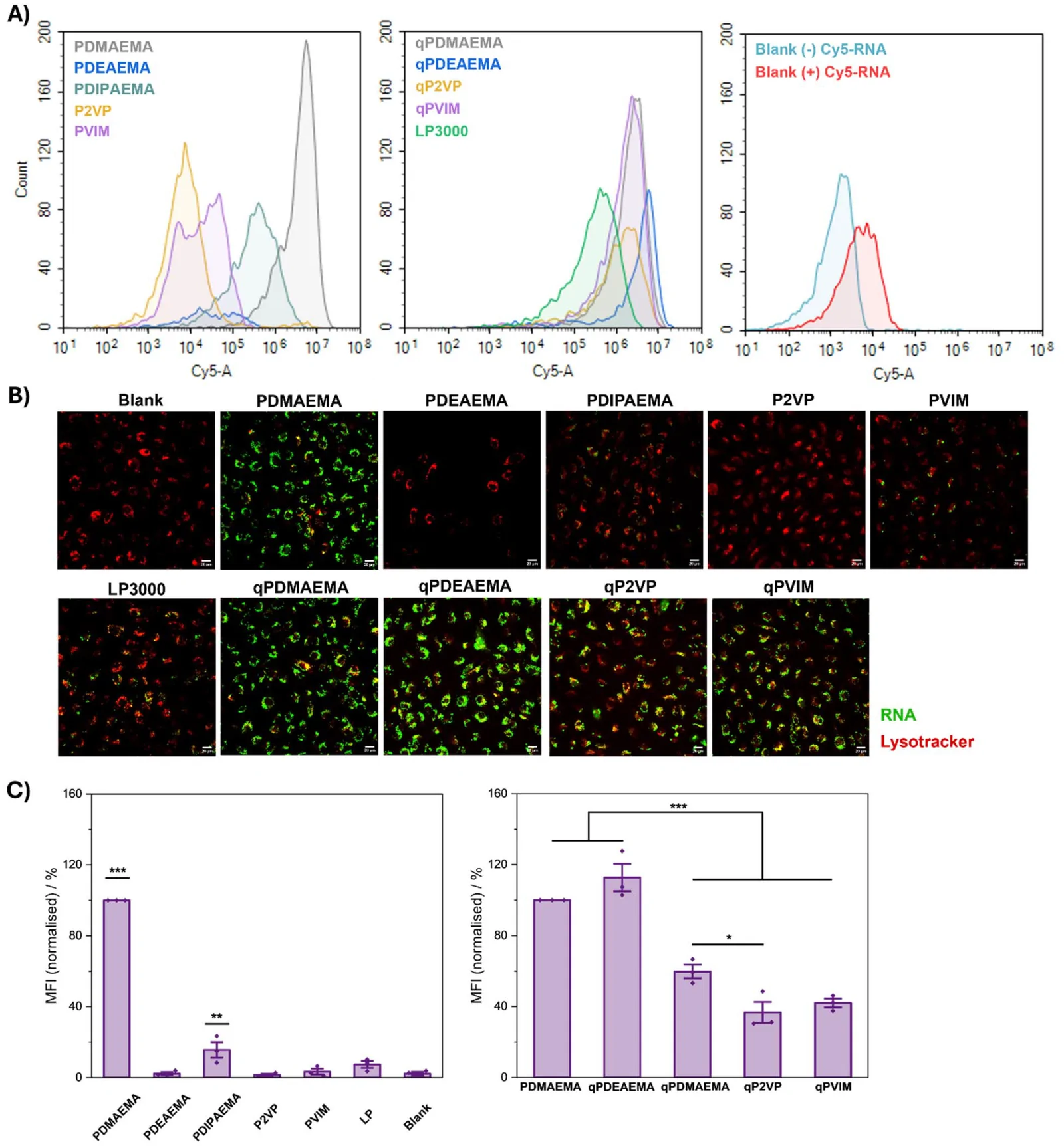
Cellular internalisation of Cy5-tagged RNA, 24 h post-transfection in HUVECs transfected with polymer brush-functionalised SiO_2_ nanoparticles (300 nm core), characterised by flow cytometry and fluorescence microscopy. (A) Flow cytometric histograms of the distribution of Cy5 fluorescence intensity/cell (gated on the Cy5+/alive cells quadrant (top left; Fig. S14); blank (+) is a transfected control using no vector. Blank (−) is a non-transfected control. (B) Corresponding representative confocal micrographs of RNA distribution and Lysotracker stainings visualised by fluorescence microscopy. (C) Median fluorescence intensity (MFI) analysis, based on flow cytometry histograms. Error bars represent s.e.m.; *N* = 3. *p < 0.05; **p < 0.01; ***p < 0.001.

Quaternisation of polymer brushes substantially increased RNA internalisation across all chemistries (Fig. 8 and Fig. S14) compared to their tertiary amine counterparts. In terms of MFI only, qPDMAEMA showed reduced internalisation relative to its tertiary amine counterpart, PDMAEMA (MFI = 60%, Fig. 8C). Indeed, this latter brush presented a shallower profile with shift towards slightly lower intensities. This is consistent with the decreased RNA binding of PDMAEMA (as shown by SPR, Fig. 4). In contrast, qPDEAEMA achieved RNA internalisation as high as that of PDMAEMA, with comparable overall profile, despite binding slightly less RNA as determined by SPR. This could suggest enhanced cellular uptake of this particular chemistry (more nanoparticles get internalised compensating for a reduced RNA binding). Most strikingly, qPVIM led to RNA internalisation at levels comparable to qPDMAEMA despite binding nearly 40-fold less RNA (based on SPR, Fig. 4). The exceptional internalisation-to-binding ratio shown by qPVIM is indeed surprising but could be associated with improved resistance to competitive desorption (Fig. 5). Importantly, all quaternised brushes displayed sharper, more uniform histogram peaks than LP3000, indicating superior delivery homogeneity (Fig. 8A).

Confocal images acquired at day 1 post-transfection (Fig. 8B) correlated well with our FACS analysis, with internalised RNA (green fluorescence) particularly evident for PDMAEMA and quaternised brushes. In addition, we observed RNA colocalised with lysosomal compartments (LysoTracker, red), appearing as yellow/orange signals in merged images. However, substantial green fluorescence was also observed in non-lysosomal compartments, indicating successful cytoplasmic delivery. Micrographs at day 3 post-transfection similarly revealed no striking differences in colocalisation between PDMAEMA and all quaternised chemistries, with quantification showing comparable Pearson correlation coefficients among these systems, all in the range of ∼0.5–0.6 (SI Fig. S15). Notably, RNA levels for qPVIM at this time point were now visually comparable to PDMAEMA. Overall, our observations suggest that the exceptional performance of qPVIM, despite its low RNA binding capacity and its capacity to promote intermediate internalisation levels, could be linked to its strong binding of RNA, slowing down desorption. Whether the formation of distinct interactomes enables superior vector trafficking remains unknown, yet we speculate that these are likely to broadly vary with the chemistry,^27,55^ perhaps leading to important differences between rigid imidazole moieties and more flexible alkyl side groups.

## Conclusions

This work establishes that the rational design of polycationic gene delivery vectors requires a mechanistic understanding of how polymer structural features independently and synergistically control RNA delivery. Our systematic investigation reveals that transfection efficiency is not determined by a single parameter, but rather by the interplay between binding, internalisation, and intracellular release, each of which considerably affected by chemical designs.

Perhaps the most significant insight is that maximizing RNA binding capacity does not necessarily yield improved performance. The success of rigid imidazole brushes, despite substantially lower maximum adsorption levels, demonstrates that transfection is governed by complex temporal dynamics where controlled release and efficient internalisation can compensate for reduced binding. It is also important to note that the N/P ratio used for transfections (set at 10 in this study, based on previous data^25^) corresponds to loading levels that are significantly below the maximum capacity of the brushes investigated here. This decoupling fundamentally challenges the conventional design paradigm that prioritises high-binding capacity.

In addition, quaternisation emerges as particularly important, not only for eliminating pH-dependence, but also for accessing a different design space that enables the modulation of binding capacities, desorption kinetics and cytotoxicity profiles. The non-monotonic relationship between hydrophobicity and toxicity, where ethyl chains are toxic but isopropyl chains are not, highlights the importance of subtle changes in the hydrophilicity–hydrophobicity balance of brushes on their conformation, the regulation of colloidal stability and interactions with cell membranes.

Importantly, our findings demonstrate effective alternatives to PDMAEMA brushes for effective knockdown of targeted proteins, across different chemistries, offering design versatility. Therefore, this expands the polycationic brush toolkit, enabling the design of vectors with tailored architectures and properties, controlling early as well as sustained silencing efficiency, with minimal cytotoxicity. The ability to independently control both magnitude and temporal dynamics of knockdown opens new possibilities for therapeutic applications requiring precise, time-dependent gene regulation. Moving forward, these design rules provide a foundation for engineering more complex architectures.

## Materials and methods

### Materials

Anhydrous acetonitrile (ACN), anhydrous *N*,*N*-dimethylformamide, 2-(dimethylamino)ethyl methacrylate (DMAEMA, *ρ* = 0.933 g mL^−1^), 2-(diethylamino)ethyl methacrylate (DEAEMA, *ρ* = 0.922 g mL^−1^), 2-(diisopropylamino)ethyl methacrylate (DIPAEMA, *ρ* = 0.900 g mL^−1^), copper chloride (Cu(i)Cl), copper bromide (Cu(ii)Br2), 2,2′-bipyridine (Bipy), anhydrous methanol (MeOH), iodomethane, anhydrous toluene, triethylamine (TEA), 1,4,8,11-tetramethyl-1,4,8,11-tetraazacyclotetradecane (Cyclam), phosphate buffered saline (PBS, 150 mM), tetrahydrofuran (THF), 1-vinyl imidazole (VIM, *ρ* = 1.039 g mL^−1^), 2-vinylpyridine (2VP, *ρ* = 0.975 g mL^−1^), were purchased from Sigma-Aldrich. Ethanol (EtOH, ≥99.8%) and isopropanol (IPA, ≥99.5%) were from Honeywell and Fisher Chemical, respectively. Dialysis tubes (3.5 kDa MWCO, BioDesignDialysis Tubing™) and hydrofluoric acid (HF, 40% v/v) were from Thermofisher. All chemicals and solvents were analytical grades unless otherwise stated. DEAEMA and DIPAEMA were purified by column chromatography with alumina. 2VP was distilled from KOH under reduced pressure (20–25 mbar, 60 °C–70 °C). Cu(i)Cl was kept under vacuum until used. Silicon wafers for ellipsometry (100 mm diameter, 〈100〉 orientation, polished on one side/reverse etched) were purchased from PI-KEM Ltd. Silicon and gold substrates were oxidised in a Henniker Plasma Cleanser (HPT-200, air plasma, 200 W for 5 min) directly prior to use. Non-functionalised dry SiO_2_ nano- and microparticles of 293 nm and 3 µm (mean diameter), respectively were purchased from Bangs Laboratories. The silane initiator, (3-trimethoxysilyl)propyl 2-bromo-2-methylpropionate, was purchased from Gelest. The thiol initiator was synthesized as previously reported.^56^ Surface plasmon resonance (SPR) chips (10 × 12 × 0.3 mm) were purchased from Ssens. Double-stranded siRNA (20 bp) (sense: ACGAUGCCUGCAGCAUUGGC; anti-sense: GCCAAUGCUGCAGGCAUCGU).

#### Cell culture reagents

Primary Human Umbilical Vein Endothelial Cells (HUVECs), pooled (C2519A) or GFP-expressing HUVECs were obtained from LONZA and Angioproteomie (cAP-0001GFP), respectively. Endothelial Cell Growth Medium 2 (EGM2) was obtained from Promocell. Opti-MEM™ I reduced serum medium (pH 7.0–7.4), Pierce™ BCA protein assay kit, trypsin, Versene (EDTA solution), Lipofectamine® 3000 (LP3000) and siRNA negative control (Silencer Select®), Hoechst 33342, live/dead assay kit and LysoTracker® Red DND-99, penicillin–streptomycin (10 000 U mL^−1^) were from Thermofisher. Hs_PECAM1_1 siRNA (sense: CAAUACACUUCACAAUUGATT; antisense: UCAAUUGUGAAGUGUAUUGGG) was purchased from Qiagen. Alexa Fluor™ 488 anti-human CD31 antibody was from BioLegend. Bovine serum albumin (BSA, heat shock fraction, *M*_w_ ∼66 kDa), DAPI (4,6-diamidino-2-phenylindole), Fluoromount™ Aqueous Mounting Medium, Cy5-tagged siRNA (MISSION® siRNA Fluorescent Universal Negative Control #1, Cyanine 5, MW = 13 849 g mol^−1^, stock concentration = 277 μg mL^−1^), Triton X-100, and PFA (paraformaldehyde) were purchased from Sigma-Aldrich.

### Deposition of ATRP initiator monolayers on silicon and gold substrates

To generate dense polymer brushes (target grafting density (*σ*) of 0.5 chains per nm^2^ (ref. 26)), a plasma oxidised silicon wafer was immersed in a solution containing 30 µL of silane initiator and 50 µL of TEA in 30 mL of anhydrous toluene. Silanisation was left to proceed overnight at room temperature (RT). Initiator-coated substrates were then rinsed with EtOH and dried with a stream of N_2_. The dry thickness of the silane initiator layer was near 1 nm, as measured *via* spectroscopic ellipsometry. Similarly, thiol initiator monolayers were deposited on oxidised gold-coated silicon substrates (for FTIR characterisation) or on SPR chips by immersion in 5 mM ethanolic solution of ω-mercaptoundecyl bromoisobutyrate, overnight, rinsed with EtOH and used within one day for synthesis.

### Synthesis of polymer brushes from flat substrates

Polymer brushes of different chemistries were synthesised *via* surface-initiated atom transfer radical polymerisation (SI-ATRP), following the corresponding ATRP recipes (Table S1) at RT (∼20 °C). All ATRP recipes, mixtures of water/alcohol^56^ and mixed halide ATRP systems of CuCl/CuBr_2_ at a ratio of 10/1 were used in this study (C–Cl bonds exhibit higher stability compared to C–Br^57,58^) to gain linearity and control over the ATRP kinetics. For hydrophobic monomers (DEAEMA, DIPAEMA, 2VP), the water component in the solvent was carefully adjusted to allow to reach 30 nm brushes in a relatively short period, compared to other works (*i.e.*, PDEAEMA and PDIPAEMA brushes of 26 and 27 nm, respectively,^59^ and 10 nm P2VP brushes,^45^ were obtained at ≥20 h), while still providing control, avoiding viscosity issues from evaporation. In a typical reaction, a solution containing CuBr_2_, ligand and monomer in 15 mL of solvent was degassed by argon bubbling for 30 min. CuCl was then rapidly added to this solution and further degassed for 15 min. Only for hydrophobic monomers (2VP, DIPAEMA, DEAEMA), the first and second degassing steps were reduced to 15 and 5 min, respectively, to limit evaporation. Initiator-coated substrates of ∼1 cm^2^ were placed in reaction vessels purged with cycles of vacuum/argon. Reaction was initiated by injecting 1 mL of polymerisation solution and stopped with vigorous addition of deionised H_2_O at different time points. Copious amounts of deionised H_2_O and EtOH were rinsed over the substrate surface. Dry polymer brush-functionalised substrates were kept in a nitrogen box until used. For the generation of quaternised polymers, the substrates were placed in a solution of 100 mM iodomethane (in DMF) for 18 h. Upon quaternisation, substrates were rinsed with EtOH and dried with N_2_.

### Synthesis of polymer brushes from SiO_2_ particles

SiO_2_ particles of diameter of 300 nm were used for all cell culture assays and chemical characterisation, except for competitive binding assays, where 3 µm particles were used instead, to allow imaging *via* epifluorescence and confocal microscopy. In both cases, polymer brush synthesis was performed under identical conditions. First, 100 mg of SiO_2_ particles were functionalised with silane initiators as follows: 100 mg of dry particles were dispersed in 1.5 mL of anhydrous toluene, with intervals of sonication. 20 µL of silane initiator and 100 µL of TEA in 500 µL of toluene were then added to the particles and left stirring for ≥18 h. Upon functionalisation, particles were washed with EtOH twice by centrifugation (4000*g* for 5 min), dried with vacuum and kept in the fridge until use. To generate brushes, initiator-functionalised particles were first redispersed in 5 mL of the corresponding ATRP solvent and degassed for 5 min. The reaction was initiated by injecting the particles solution to a concentrated ATRP solution (prepared with quantities for 15 mL of solvent, but dissolved in 10 mL), while stirring and stopped at the time required to achieve 30 or 20 nm target thickness (as determined from the polymerisation kinetics on flat substrates, see Table S1). The reaction was stopped by dilution (1 : 5) in oxygenated deionised H_2_O and under vigorous air bubbling until the solution turned blue. Brush-functionalised SiO_2_ particles were washed with EtOH twice (or acetonitrile in the case of P2VP) by centrifugation, dried under vacuum and kept in the fridge until use.

### Thermogravimetric analysis (TGA)

The mass of the polymer brush grafted on nanoparticles was determined by TGA using a Thermogravimetric Analyzer TGA 5500 (TA Instruments). ∼5 mg of dry polymer brush-functionalised SiO_2_-nanoparticles were heated at a heating rate of 10 °C min^−1^ from RT to 950 °C under N_2_ at a constant flow of 25 ml min^−1^. An estimation of the brush thickness was done using eqn (1).^26^ The TGA traces of initiator-functionalised nanoparticles were used to account for the initiator and volatile components in the calculation (accounting for nearly 1% the total mass).1
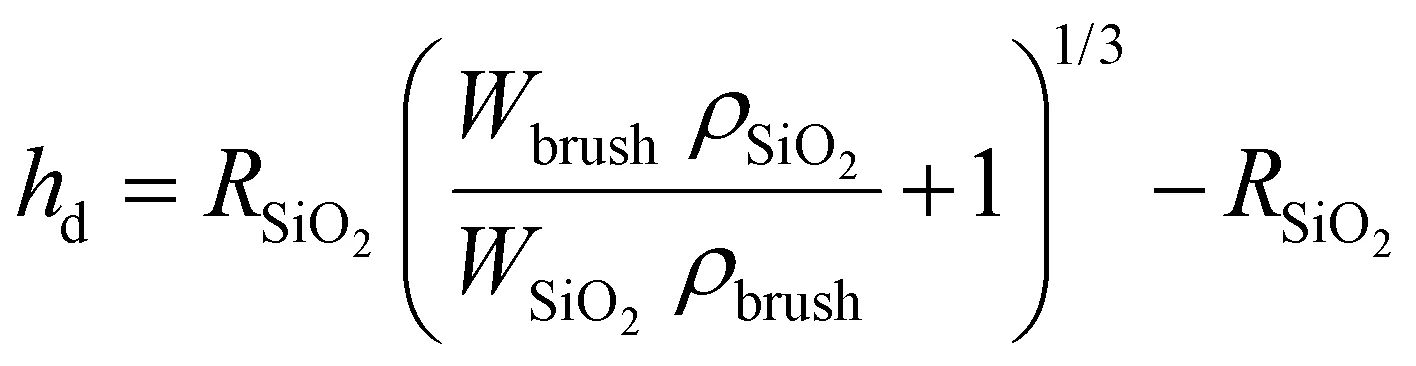


Estimation of the polymer brush dry thickness based on TGA profiles. *W*_brush_ and *W*_SiO_2__ are the mass of polymer brush and nanoparticles, respectively. *R*_SiO_2__ is the radius of a particle, and *ρ*_SiO_2__ and *ρ*_brush_ is the density of the corresponding polymer chemistry.

### Quaternisation of polymer brushes from SiO_2_ particles

10 mg of polymer brush-functionalised substrates were dispersed in iodomethane (100 mM in DMF) for 18 h. Upon quaternisation, particles were washed with EtOH twice by centrifugation, dried under vacuum and kept in the fridge until use.

### Determination of molecular weight and brush grafting densities

To calculate the grafting density of polymer brushes generated from silicon substrates, brushes were degrafted from silica nanoparticles by etching with HF, and the molecular weight of these polymer chains was determined by gel permeation chromatography (GPC). Hydrofluoric acid is extremely hazardous. All HF handling was performed in a fume hood with appropriate personal protective equipment. Briefly, 2 mL of hydrofluoric acid solution (40% v/v) was added dropwise to an equal volume of SiO_2_-PDMAEMA suspension (20 mg mL^−1^ in deionised H_2_O) in appropriate polypropylene tubes and left to incubate for 1 h at RT. Gentle mixing was applied to ensure complete silica dissolution. The solution was then carefully neutralised by dropwise addition of excess NaOH (2 M) and transferred to a 3.5 kDa dialysis tube, followed by dialysis against deionised H_2_O overnight, repeated twice. The purified material was freeze-dried. GPC measurements were performed using an Agilent 1260 Infinity system equipped with PLgel columns, a refractive index detector (RID) and autosampler, operating in THF (HPLC grade) at 25 °C, and calibrated with poly(methyl methacrylate) standards ranging from 500 to 2 200 000 Da [Mark–Houwink parameters for PMMA (*K* = 18.32 × 10^−5^ dL g^−1^, *α* = 0.69)]. Polymers were thoroughly dissolved in THF at a concentration of 1.25 mg mL^−1^ and filtered through a 0.2 µm PTFE filter prior to analysis. Molecular weights were extracted and reported as PMMA equivalents. The grafting density (*σ*) was calculated using eqn (2):^26^2
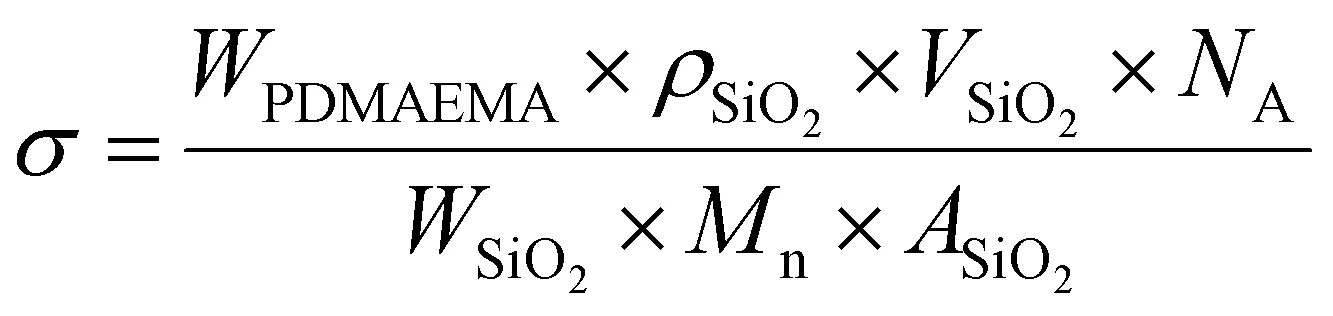


Estimation of the grafting density of polymer brushes cleaved from SiO_2_ nanoparticles. *W*_PDMAEMA_ is the weight loss percentage of PDMAEMA and *W*_SiO_2__ is the residual weight percentage obtained from TGA. *ρ*_SiO_2__ is the density of silica (2.4 g cm^−3^), *V*_SiO_2__ is the volume of silica nanoparticles 
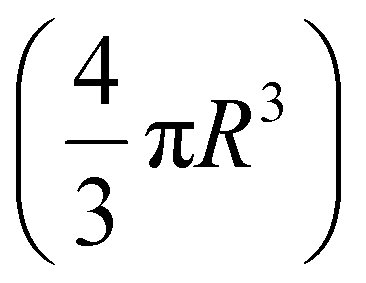
, *A*_SiO_2__ is the surface area of silica nanoparticles (4π*R*^2^), *R* is the radius of silica nanoparticles (146.5 nm), *N*_A_ is Avogadro's number (6.022 × 10^23^ mol^−1^) and *M*_n_ is the number-average molecular weight from GPC.

### Dynamic light scattering (DLS)

Size distribution and *ζ*-potential of polymer brush-functionalised nanoparticles were characterised with a Zetasizer Nano ZS (Malvern), at 25 °C. All samples were taken from the same batches used for transfection assays and diluted to 0.1 mg mL^−1^ in PBS (1×; pH 7.4, 150 mM). In addition, diluted PBS (0.1×; diluted with deionised H_2_O; final ionic strength: 15 mM) was used to reduce the effect of ionic strength on *ζ*-potential measurements.

### X-ray photoelectron spectroscopy (XPS) and Fourier transform infrared spectroscopy (FTIR)

Surface chemical characterisation of polymer brushes from flat silicon substrates and SiO_2_ nanoparticles was assessed by XPS using a Nexsa Spectrometer (ThermoFisher) with monochromatic Al Kα X-ray radiation. Survey and high-resolution spectra were collected with analyser pass energy set as 200 eV (1 eV energy resolution, 50 scans) and 50 eV (0.1 eV energy resolution, 10 scans), respectively. Each sample was characterised in three different spots. Prior analysis of the spectra, charge correction was performed with adventitious carbon as a reference at 284.8 eV. For characterisation of SiO_2_ nanoparticles, a 10 µL drop of nanoparticles suspension (at 10 mg mL^−1^) was deposited on gold-coated silicon substrates and dry under vacuum before analysis.

FTIR spectra of polymer brushes from flat substrates and nanoparticles were obtained using a Bruker Tensor 27 spectrometer. To characterise polymer brushes generated from gold substrates, an Advanced Grazing Angle specular reflection accessory (Pike Technologies) at a fixed incidence angle of 80° was used to reflect the IR light. The reflected light was collected with a liquid nitrogen-cooled MCT detector. Measurements were taken at a resolution of 4 cm^−1^ with 16 scans in the range of 600–4000 cm^−1^. A gold coated-mirror was used to subtract the background.

### Study of swelling behaviour *via* ellipsometry

Ellipsometry measurements were carried out with an α-SE instrument (J. A. Woollam) at an incidence angle of 70° over a 380–900 nm multi-wavelength spectral range. Psi and delta spectroscopic curves were extracted and fitted against simple native oxide/Cauchy models in CompleteEASE (J.A. Woollam). The dry thickness was determined in air, whereas for the determination of the swollen thickness, substrates were placed in an in-house-built liquid cell fitted with quartz windows normal to the light beam path. Substrates were left to equilibrate for 15 min in deionised H_2_O and PBS (at pH 7.4, and 5.5 or 4.0, to enabling protonation matching the reported p*K*_a_ values). All measurements were carried out in triplicates using substrates from separate polymerisation solutions, and at RT.

### Study of nucleic acids binding kinetics of polymer brushes *via* surface plasmon resonance (SPR)

Polymer brush-functionalised SPR chips were prepared in the same way as for silicon substrates. SPR assays were carried out on a Biacore 1K+. The flow rate of injections and running buffer was maintained constant throughout the experiment at a value of 10 µL min^−1^. A change of 10^4^ RU corresponding to 1 µg cm^−2^ of binding capacity was considered based on the literature.^60^ Brush-functionalised SPR chips were mounted on plastic sensor chip supports, docked, and left to equilibrate with the respective running buffer until a stable baseline was obtained (normally, within 45 min). For each nucleic acid material, a set of concentrations ranging from 1 to 20 µg mL^−1^ was prepared by serial dilutions, with the buffer matching the running buffer to reduce RI drifts. All injections were carried out incrementally, starting from the lowest concentration. Upon equilibration, 50 µL of nucleic acid solutions were injected for 5 min, followed by a washing step with running buffer for 10 min. Prior to each subsequent injection, regeneration with 2 M NaCl and further equilibration were performed.

### Competitive binding assays

We characterised the competitive desorption of Cy5-tagged siRNA from PDMAEMA, qPDMAEMA, qPDEAEMA and qPVIM functionalised-SiO_2_ microparticles (3 µm) in the presence of full cell lysates from HUVECs (obtained from three different culture batches). To generate cell lysates, a confluent cell layer (cultured in the same conditions described below) was washed twice with 2 mL of ice-cold PBS containing protease (0.05%) and phosphatase inhibitors (20 µL NaF and 100 µL Na_3_VO_4_ per 10 mL of PBS, pH 7.4), lysed by introducing 0.5 mL of lysis buffer (8 M urea in 20 mM HEPES, pH 8.0; Na_3_VO_4_, 100 mM; NaF, 500 mM; β-glycerol phosphate, 1 M; Na_2_H_2_P_2_O_7_, 250 mM) onto the layer, followed by gentle scraping using a cell scraper. The cell suspension was then transferred to an Eppendorf Protein Lo-bind tube and further lysed by probe sonication at 50% intensity for 10 s, twice, with a 10 s rest period in between. The vial containing the cell suspension was kept on ice during sonication to prevent heat-induced degradation of phosphoproteins. The protein concentration of the generated lysates was found to be 2.5 ± 0.4 mg mL^−1^ (assessed by Pierce BCA protein assay). Cell lysates were stored at −80 °C until use. Competitive binding assays were performed based on the literature,^27^ with some modifications. A PDMS chamber with circular wells (diameter ∼4 mm) with a glass bottom was used. Briefly, 20 µL of brush-functionalised SiO_2_ microparticles (at 500 µg mL^−1^) were placed into each well and allowed to adsorb to the glass for 10 min. 20 µL of Cy5-tagged siRNA (3 µg mL^−1^) in PBS were then added to the wells to assemble the complexes, for 30 min at RT, at a final N/P of 10 (as described in the next section “Gene knockdown assays”). After complexation, the wells were washed twice with PBS (aspirating 30 µL each time) before adding 30 µL of cell lysate. The brush-RNA complexes were imaged with a Nikon CSU-W1 SoRa spinning disk confocal microscope at 37 °C before lysate injection (*t*_0_) and during 24 h. At least 100 microparticles per image were used, at comparable densities. Quantitative analysis of the RNA desorption was done in ImageJ Fiji by extracting the mean intensity of all microparticles at each time point using Stardist,^61^ and normalised to intensities at t_0_. The corresponding background was subtracted every time.

### Gene knockdown assays

The library of brush chemistries was tested for its ability to knock down (KD) the expression of CD31 (PECAM-1) in adherent cell culture monolayers over time. HUVECs were cultured in T75 flasks at 37 °C under 5% CO_2_, in EGM2 media (containing 100 U mL^−1^ of penicillin–streptomycin). Once cells reached a >90% confluent monolayer, cells were seeded on glass cover slips (in 24 well plates). All experiments were conducted using cells at passage ≤6. Cell detachment was performed with a trypsin/Versene dissociation solution (1 : 9 v/v). HUVECs were seeded and left to incubate for 48 h (to enable complete and homogenous PECAM-1 expression), at a starting cell density (cells per well) of 50 000 cells per well and 25 000 cells per well to characterise the KD efficiency after 1- and 3-days post-transfection incubation, respectively. For the evaluation of the long-term KD efficiency (for 7, 10 and 14 days), the starting seeding density was 13 000 cells per well. Prior to transfection, cells were washed with pre-warmed dPBS and Opti-MEM medium, once, and incubated in 400 µL of Opti-MEM for 30 min. To produce the RNA–polymer brush complexes, brush-functionalised nanoparticles were first redispersed in PBS pH 7.4 (with exception of PDIPAEMA and P2VP, where the pH was adjusted to 5.5 and 4, respectively to allow dispersion), at a concentration of 10 mg mL^−1^. Brush-SiO_2_ nanoparticles and siRNA (20 µM) solutions were then diluted separately in 50 µL of PBS, (in quantities required for an N/P of 10, according to eqn (3)), and then mixed gently by pipetting up and down. Complexation was allowed to proceed for 30 min. For the positive control, LP3000 (1.25 µL) was used following the supplier instructions and using the same amount of siRNA as for brush complexes. A scrambled non-targeting siRNA was used as negative control, complexed with LP3000 or polymer brush-SiO_2_ nanoparticles.3
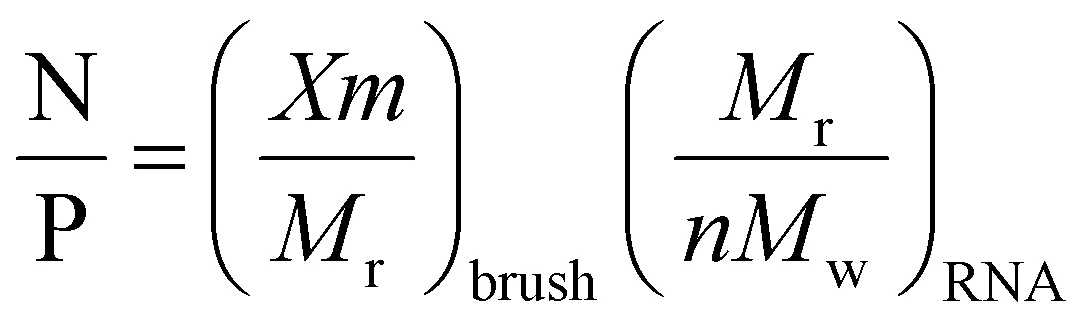


Ratio of nitrogen from polycations to phosphate from the RNA backbone (N/P ratio). *X* is the polymer brush mass fraction determined *via* TGA, *m* is the mass of brush-functionalised SiO_2_ nanoparticles, *M*_r_ brush is the molar mass of the brush repeating unit (*e.g.*, DMAEMA = 157.2 g mol^−1^), *M*_r_ RNA is the molar mass of a ribonucleotide (∼319.14 g mol^−1^), *n* is the moles of RNA (siRNA = 25 pmol) and *M*_w_ is the molecular weight of the RNA strand (siRNA of 20 bp = 12 765.72 g mol^−1^). *M*_w_/*M*_r_ RNA ratio accounts for the number of phosphate groups per RNA molecule.

100 µL of solution of RNA–polymer brush complexes was then added to each well dropwise and mixed by gently shaking (final siRNA concentration of 50 nM per well) and left to incubate for 4 h at 37 °C. Following the initial incubation, the media was replaced by 500 µL of EGM2 for further incubation time. Finally, cells were washed twice with PBS and fixed with ice-cold PFA (4% in PBS, 10 min). To assess PECAM-1 knockdown, cells were first permeabilised with Triton X-100 (0.2% in PBS) and blocked for 1 h in blocking buffer prior to staining with anti-CD31 (1 : 200) and DAPI (5 µg mL^−1^) solution (in blocking buffer). Samples were left to incubate in the fridge overnight and then washed twice with PBS. Coverslips were mounted on glass coverslips in Fluoromount™ media and imaged with a Leica DMi8 epifluorescence microscope. Quantitative analysis of the KD efficiency was done in ImageJ Fiji by comparing the intensities of each treatment against LP3000 negative control.

### Viability assays

To assess the toxicity of polymer brush-siRNA complexes (N/P of 10), cells were stained for live/dead and nuclei (with Hoescht, at 1 µg mL^−1^) 1-day post-transfection, for 25 min at 37 °C in EGM2 media. The staining solution was then replaced with EGM2 media and cells were imaged with a Leica DMi8 epifluorescence microscope. Quantitative analysis of the cell death rate and densities was done in ImageJ Fiji. The dead cells ratio was calculated as the ratio of dead cells against the total number of cells (stained nuclei). Cell densities were extracted from stained nuclei and normalised to those on the non-transfected controls.

### RNA cellular internalisation and colocalisation studies

The ability of polymer brush-functionalised SiO_2_ nanoparticles to internalise RNA was characterised by flow cytometric analysis (FACS) using an ACEA Novocyte 3000 analyser. HUVECs were transfected with Cy5-tagged RNA following the same procedure above described and analysed at 24 h post-transfection. Cells were detached from coverslips with a dissociation solution (EDTA/trypsin) and washed with 50 µg mL^−1^ of a heparin solution once. The pellet was collected by centrifugation (200*g* for 5 min, at 4 °C) and resuspended in 400 µL of ice-cold FACS buffer (composition: 1% BSA, 2 mM EDTA and 1 : 1000, 5 µg mL^−1^ of DAPI, in PBS) and kept in ice until FACS analysis (within 1 h). Data acquisition and analysis, including gating strategies, were performed using NovoExpress Software. Median Fluorescence intensity (MFI) was normalised to PDMAEMA. In a separate 24-wells plate, HUVECs transfected with Cy5-tagged RNA were stained with LysoTracker® Red DND-99 at 50 nM and Hoechst (1 µg mL^−1^) for 1 h at 37 °C. Upon incubation, cells were washed with pre-warm dPBS once, and fixed with PFA (4% in PBS, 10 min). Coverslips were then mounted on glass coverslips in Fluoromount™ and imaged with a Nikon CSU-W1 SoRa spinning disk confocal microscope. Cultures were also stained for Lysotracker to determine endosomal escape efficiencies at 3 days post-transfection. Cy5 and Lysotracker signal were extracted to study co-localisation through Pearson correlation coefficients using JACoP plugin in ImageJ Fiji.

### Statistical analysis

All statistical analyses were conducted using OriginPro (9.95/10.3). Data from independent experiments (*N*) are presented as means ± s.e.m., while data from individual measurements (*n*) are presented as means ± s.d. For comparisons, one-way ANOVA was performed, followed by Tukey's HSD *post hoc* test for pairwise comparisons when significant differences were detected. Statistical significance was defined as *p* < 0.05. Significance levels are indicated as *p < 0.05, **p < 0.01, ***p < 0.001.

## Conflicts of interest

The authors declare no conflicts of interest.

## Supplementary Material

BM-014-D6BM00813E-s001

## Data Availability

Data associated with this manuscript, including source data files, quantified data and microscopy images will be deposited on the publicly accessible Queen Mary Research Online (QMRO) repository, or available directly from the authors upon request. Supplementary information (SI): additional ellipsometry, FTIR, XPS and SPR data, as well as additional confocal and flow cytometry characterisation of siRNA delivery and silencing performance. See DOI: https://doi.org/10.1039/d6bm00813e.
