# Supplementary material for: Impact of the physico-chemical properties of surface-initiated polymer brushes on siRNA binding and transfection efficiency
Source: Biomater Sci. 2026 Sep 22;14(22):5892–911. doi: 10.1039/d6bm00813e (PMC13639890; doi:10.1039/d6bm00813e)
Supplement: Supplementary file 2 [file BM-014-D6BM00813E-s001.pdf]

Supplementary Information (SI) for Biomaterials Science.
This journal is © The Royal Society of Chemistry

Impact of the Physico-Chemical Properties of Surface-Initiated Polymer brushes on siRNA Binding and Transfection Efficiency

Supporting information

Carlos E. Neri-Cruz[1], Davide Carta[1], and Julien E. Gautrot[1*]

[1] School of Engineering and Materials Science, Queen Mary University of London, Mile End Road, London E1 4NS, United Kingdom.

* Correspondence:

Julien E. Gautrot (j.gautrot@qmul.ac.uk)

**Brush chemistry**	**Solvent**	**Monomer**	**Ligand**	**$CuBr_2$**	**CuCl**	**Target thickness / reaction time**
PDMAEMA	H_2O:EtOH 7:3	21.0 mmol (3.30 g)	2,2′-Bipyridine 1025 µmol (160 mg)	40.3 µmol (9 mg)	419 µmol (41.5 mg)	30 nm / 22 min
PDEAEMA	MeOH:H_2O 6.5:3.5	16.5 mmol (3.06 g)	2,2′-Bipyridine 807 µmol (126 mg)	31.8 µmol (7.1 mg)	330 µmol (32.7 mg)	30 nm / 85 min
PDIPAEMA	IPA:H_2O 6.5:3.5	16.5 mmol (3.53 g)	2,2′-Bipyridine 807 µmol (126 mg)	31.8 µmol (7.1 mg)	330 µmol (32.7 mg)	30 nm / 30 min
P2VP	ACN/H_2O 7:3	31.2 mmol (3.30 g)	Cyclam 861 µmol (220.8 mg)	40.3 µmol (9 mg)	419 µmol (41.5 mg)	30 nm / 17.5 min
PVIM	H_2O	31.6 mmol (2.97)	Cyclam 987 µmol (253.1 mg)	40.3 µmol (9 mg)	419 µmol (41.5 mg)	20 nm / 22 h

Table S1. *Summary of ATRP recipes for each brush chemistry, in 15 mL of solvent. A 10/1 ratio of Cu(I)/Cu (II) was kept constant.*

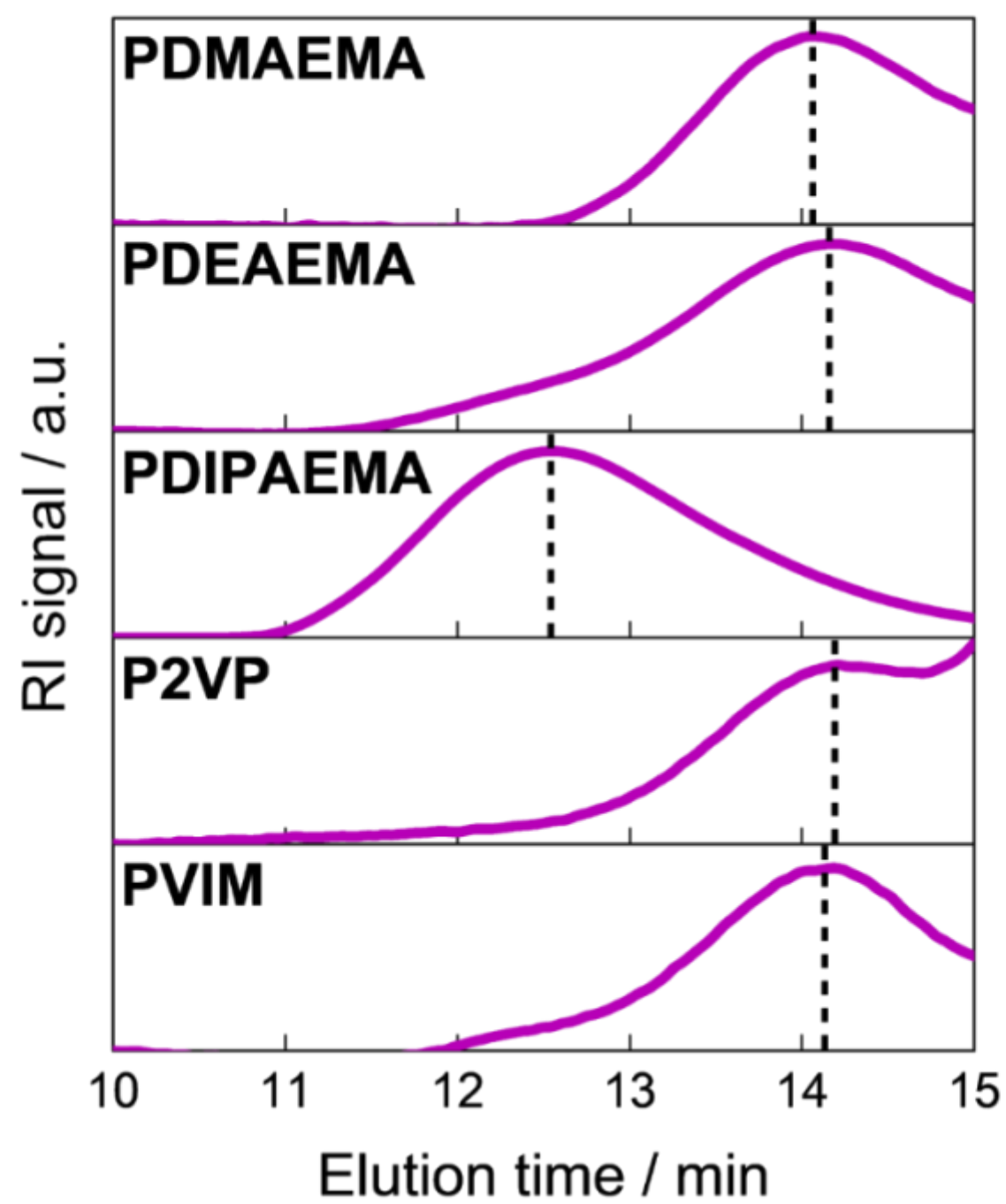

Brush chemistry	Mp (g/mol)	Mn (g/mol)	Mw (g/mol)	Đ Mw / Mn	σ (chains/nm^2)
PDMAEMA	83849	61758	92889	1.5	0.47
PDEAEMA	74399	59086	143316	2.4	0.39
PDIPAEMA	424762	202614	462187	2.3	0.09
P2VP	73297	79176	97658	1.2	0.40
PVIM	28775	64584	108315	1.7	0.29

Figure S1. *GPC characterisation of the molecular weight distributions of polymer brushes cleaved from SiO_2 nanoparticles (300 nm core). Mn values were used to estimate grafting densities (σ) shown in the last column. Measurements were performed in THF at 25°C using poly(methyl methacrylate) standards for calibration. Vertical dashed lines indicate peak maximum elution times. Low molecular weight fractions (~1000 g/mol, t ~18 min), including non-initiated silane initiators, were not considered in the peak integration (note that this shoulder was broader for P2VP, extending to t ~15 min).*

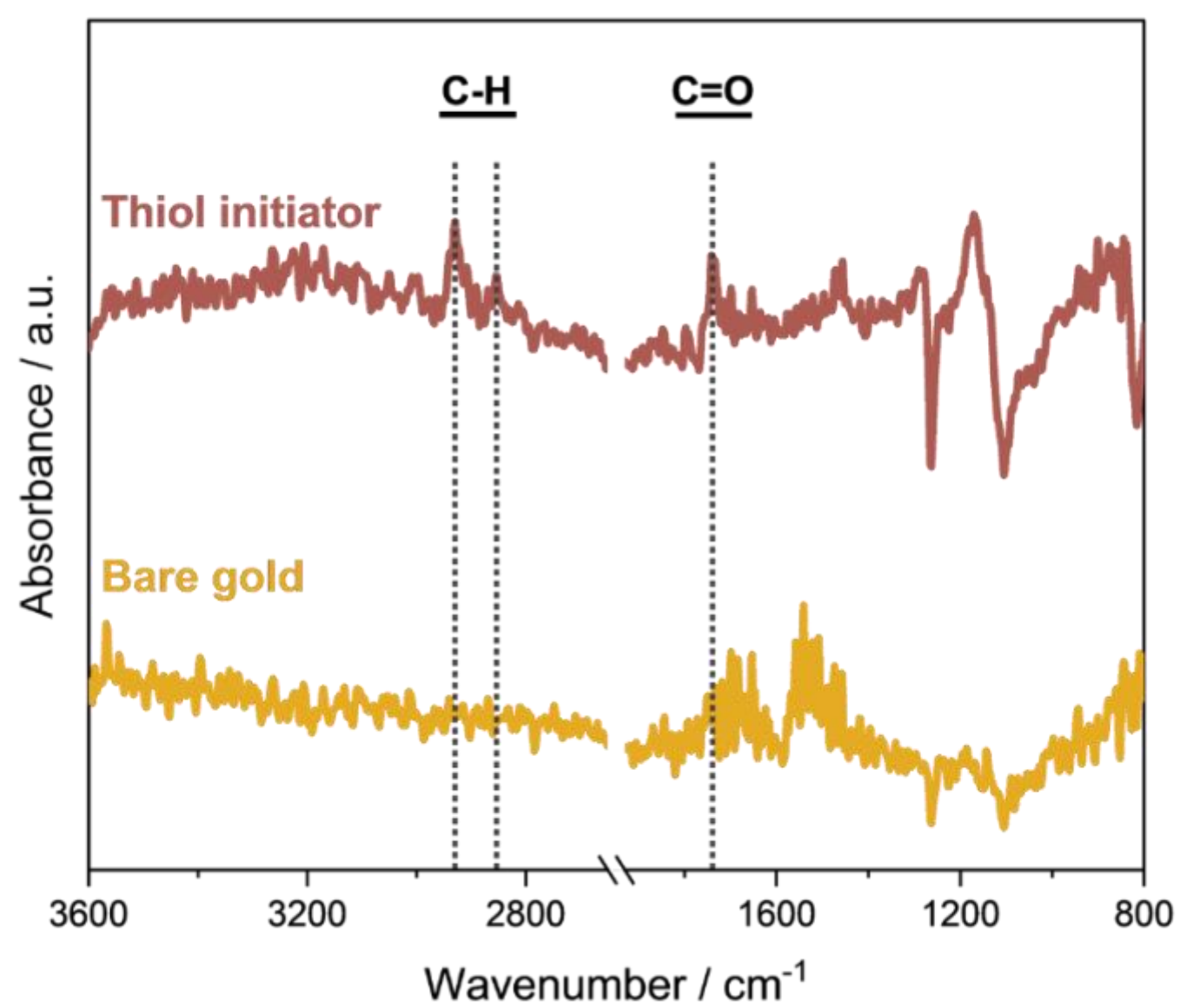

Figure S2. *FTIR spectra of gold-coated silicon substrates confirming the deposition of ATRP thiol monolayers by the appearance of C-H stretch (2930 and 2854 cm^{-1}) and C=O (1735 cm^{-1}) bands, in good agreement with the literature [45].*

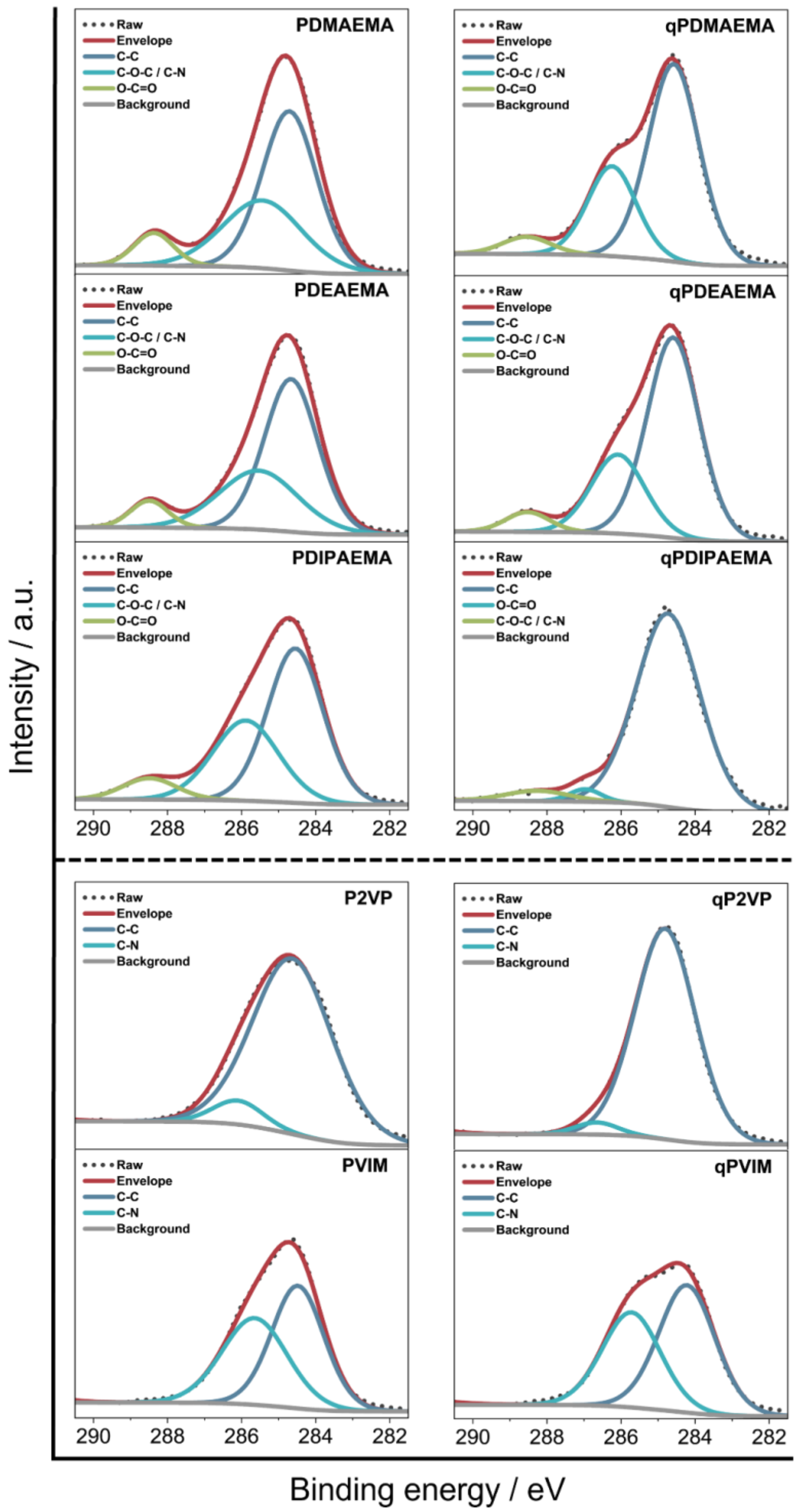

Figure S3. *High-resolution XPS analysis of C1s in tertiary and quaternary amine-bearing polymer brushes generated from SiO_2 nanoparticles (300 nm core).*

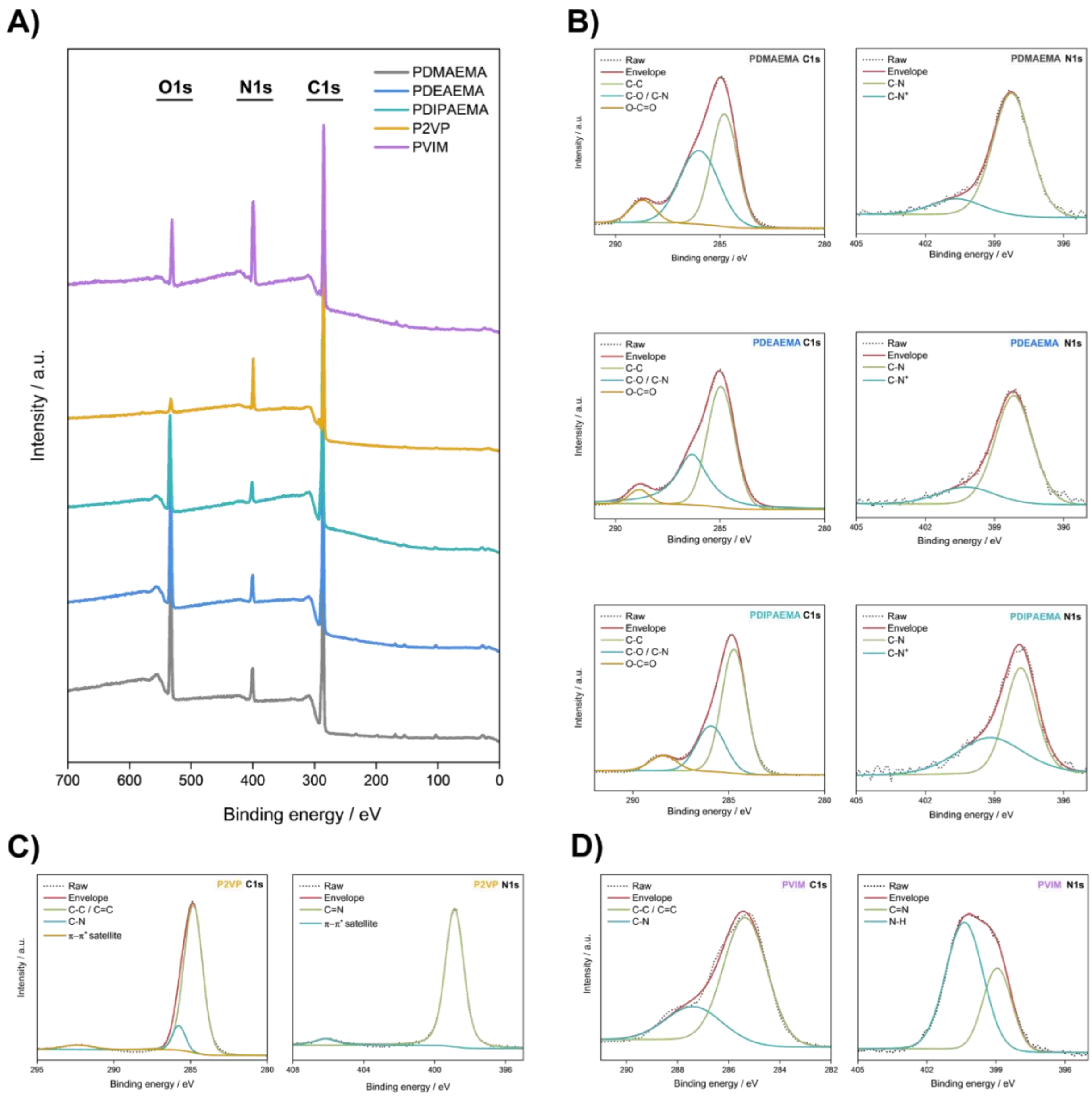

Figure S4. *XPS analysis of tertiary amine-bearing polymer brushes generated from silicon wafers. High-resolution spectra and deconvolution of **A)** alkyl amine- and **B)** heterocyclic amine-bearing brushes at the C 1s and N1s levels. C) Corresponding surveys. Charge correction was performed with adventitious carbon as a reference at 284.8 eV.*

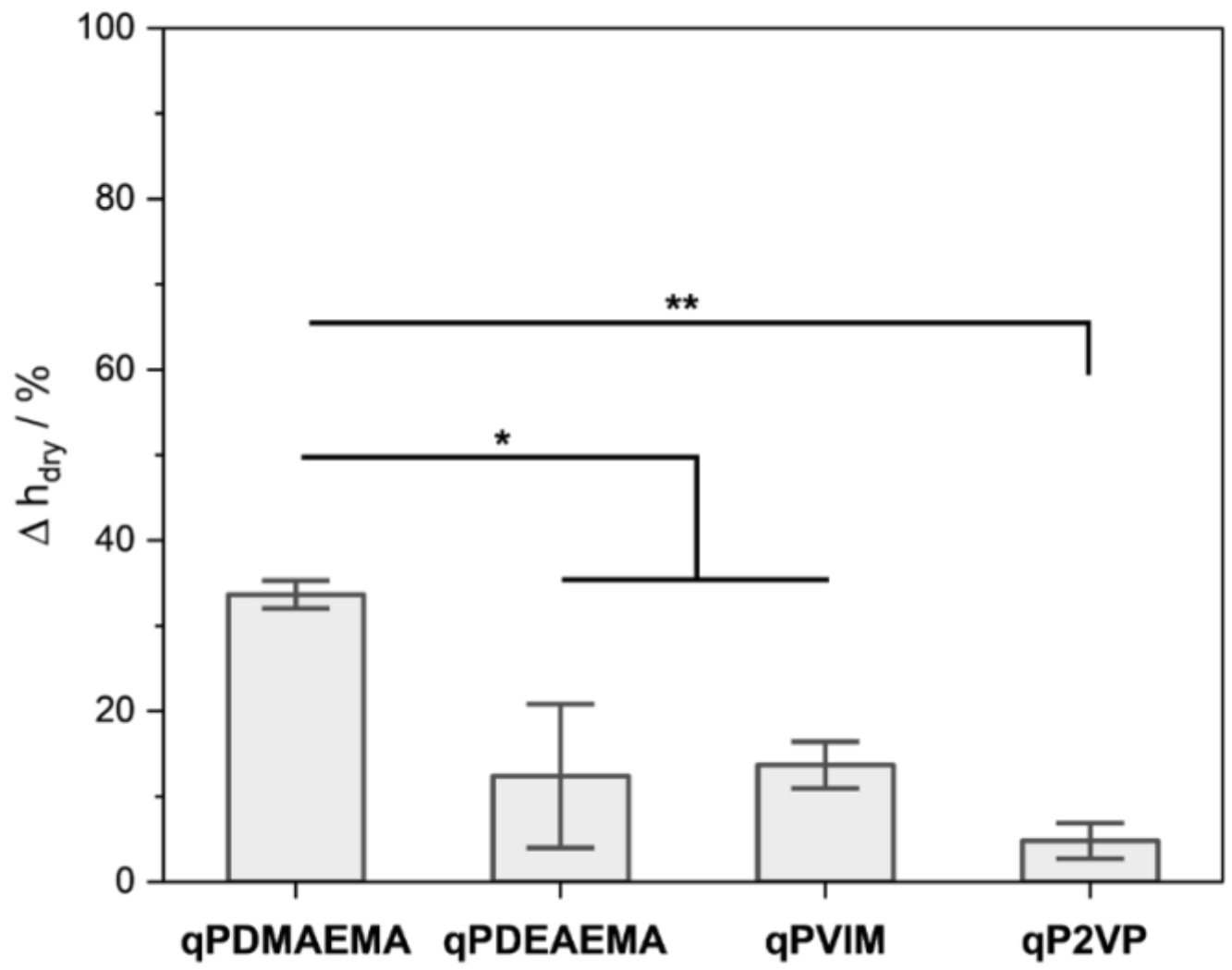

Figure S5. *Changes in the brush dry thickness (h_{dry}) upon quaternisation with methyl iodide. Means ± standard errors. N = 3. *p < 0.05; **p < 0.01; ***p < 0.001.*

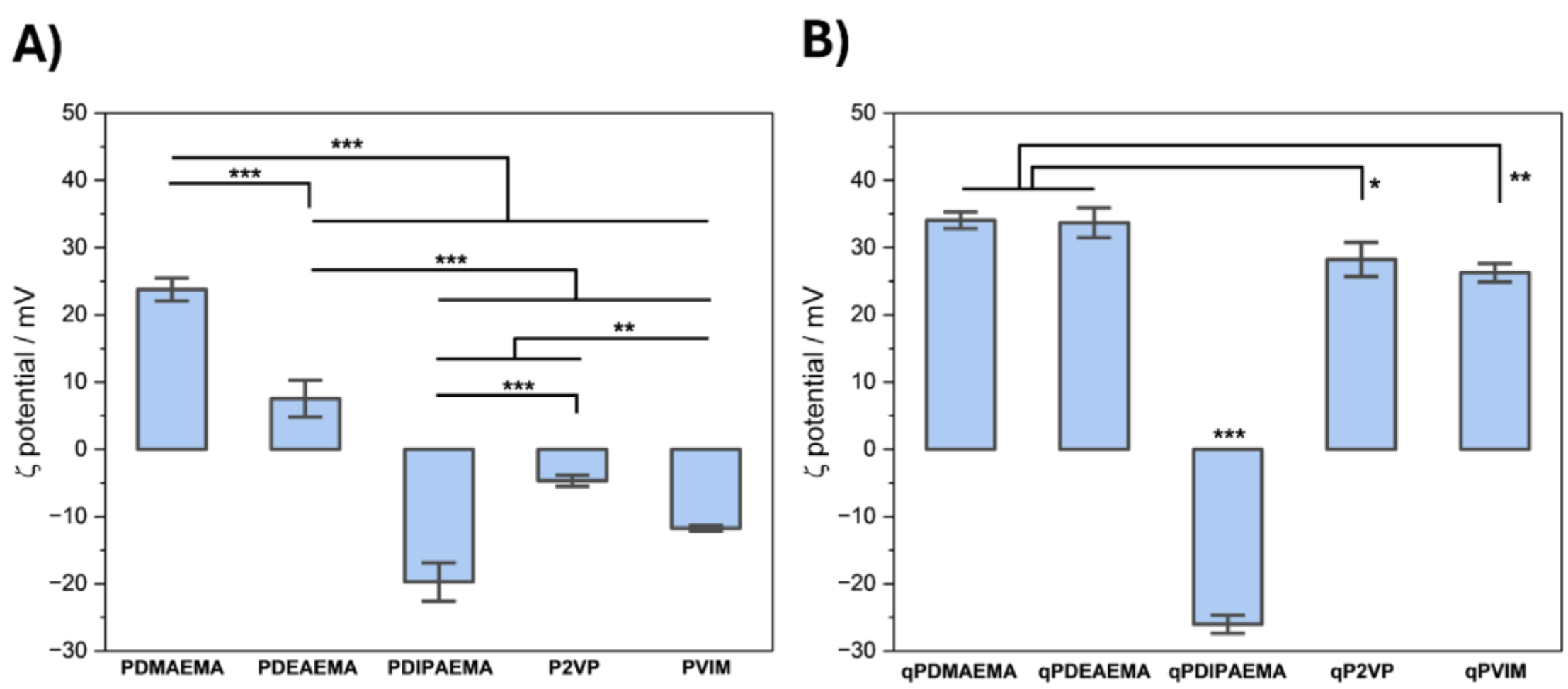

Figure S6. *Colloidal stability of polymer brush-functionalised SiO_2 nanoparticles (300 nm core) at low ionic strength by DLS. ζ potentials of* ***A)*** *tertiary amines and* ***B)*** *quaternary ammoniums were determined in diluted PBS (15 mM, in deionised H_2O, without pH adjustment) at 25°C. Error bars represent s.d; N=3 . *p < 0.05; **p < 0.01; ***p < 0.001.*

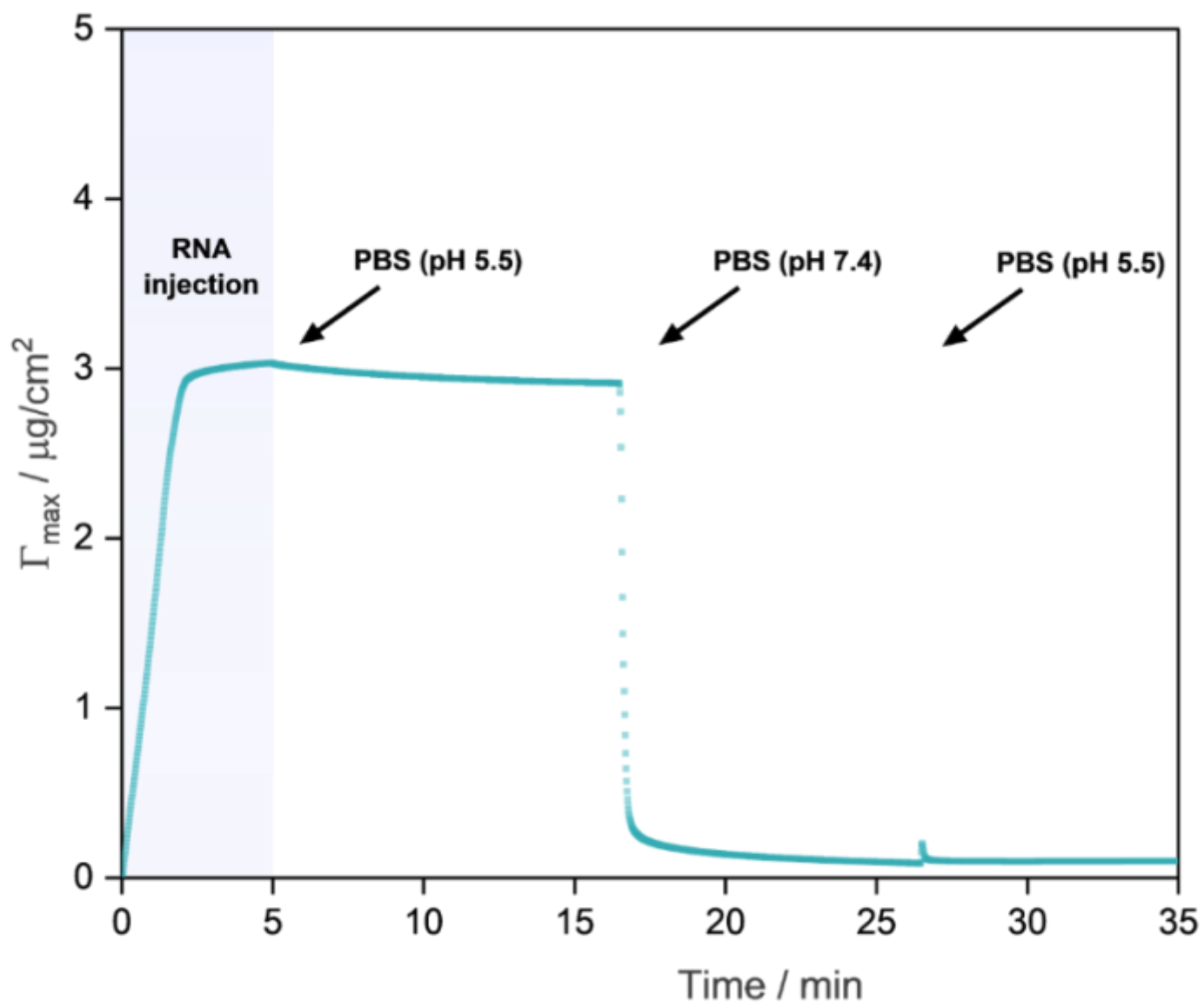

Figure S7. *Impact of pH responsiveness on desorption of siRNA (20bp) from PDIPAEMA brushes (pKa ~ 6) by SPR, at 25°C. The SPR trace shows injection with rapid adsorption of siRNA (20 µg/mL in PBS, pH 5.5), followed by a washing step prior to switching to neutral pH (PBS, pH 7.4), where most of the siRNA is released.*

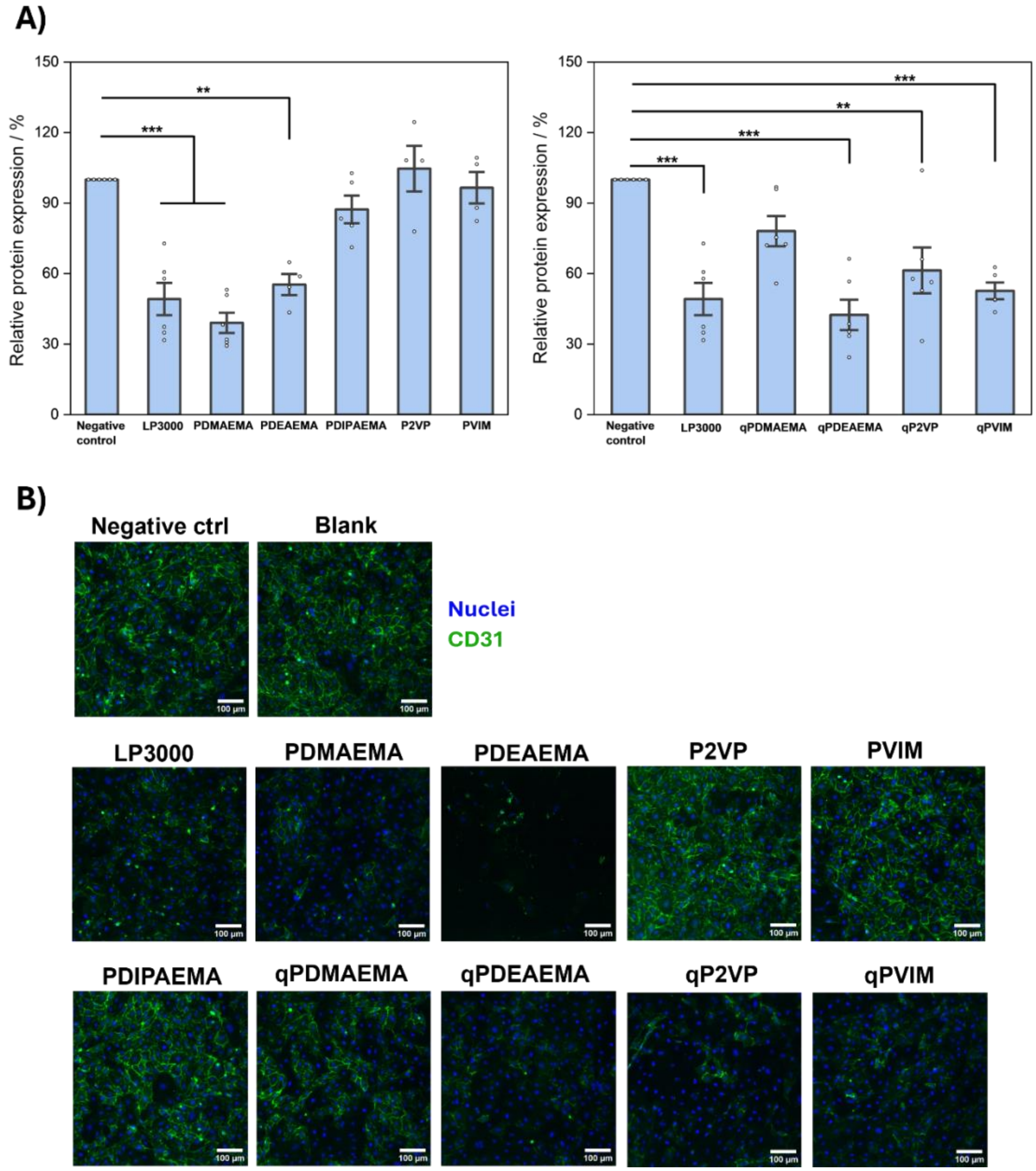

Figure S8. *PECAM-1 (CD31) knockdown efficiency in HUVECs at day 1 post-transfection with polymer brush-functionalised* SiO_2 *nanoparticles (300 nm core).* ***A)*** *Quantification of relative protein expression normalised to LP3000 negative control.* ***B)*** *Representative immunofluorescence images showing CD31 expression (green) and nuclear staining (blue). Error bars represent s.e.m.;* $N \geq 4$. *$^*p < 0.05$; $^{**}p < 0.01$; $^{***}p < 0.001$).*

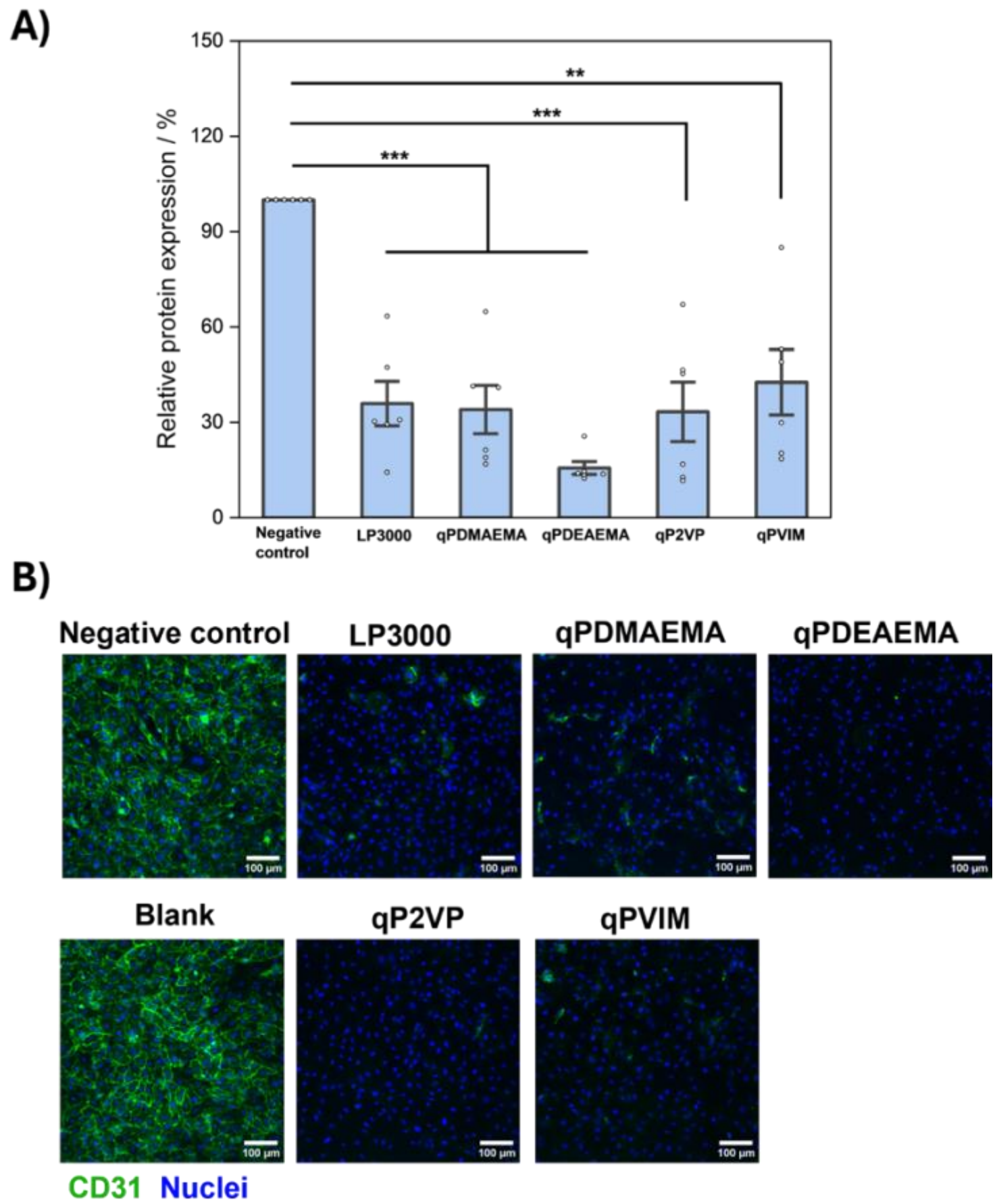

Figure S9. *PECAM-1 knockdown efficiency in HUVECs at day 3 post-transfection with polymer brush-functionalised SiO_2 nanoparticles (300 nm core).* ***A)*** *Quantification of relative protein expression normalised to LP3000 negative control.* ***B)*** *Representative immunofluorescence images showing CD31 expression (green) and nuclear staining (blue). Error bars represent s.e.m.; $N \geq 4$. *$p < 0.05$; **$p < 0.01$; ***$p < 0.001$.*

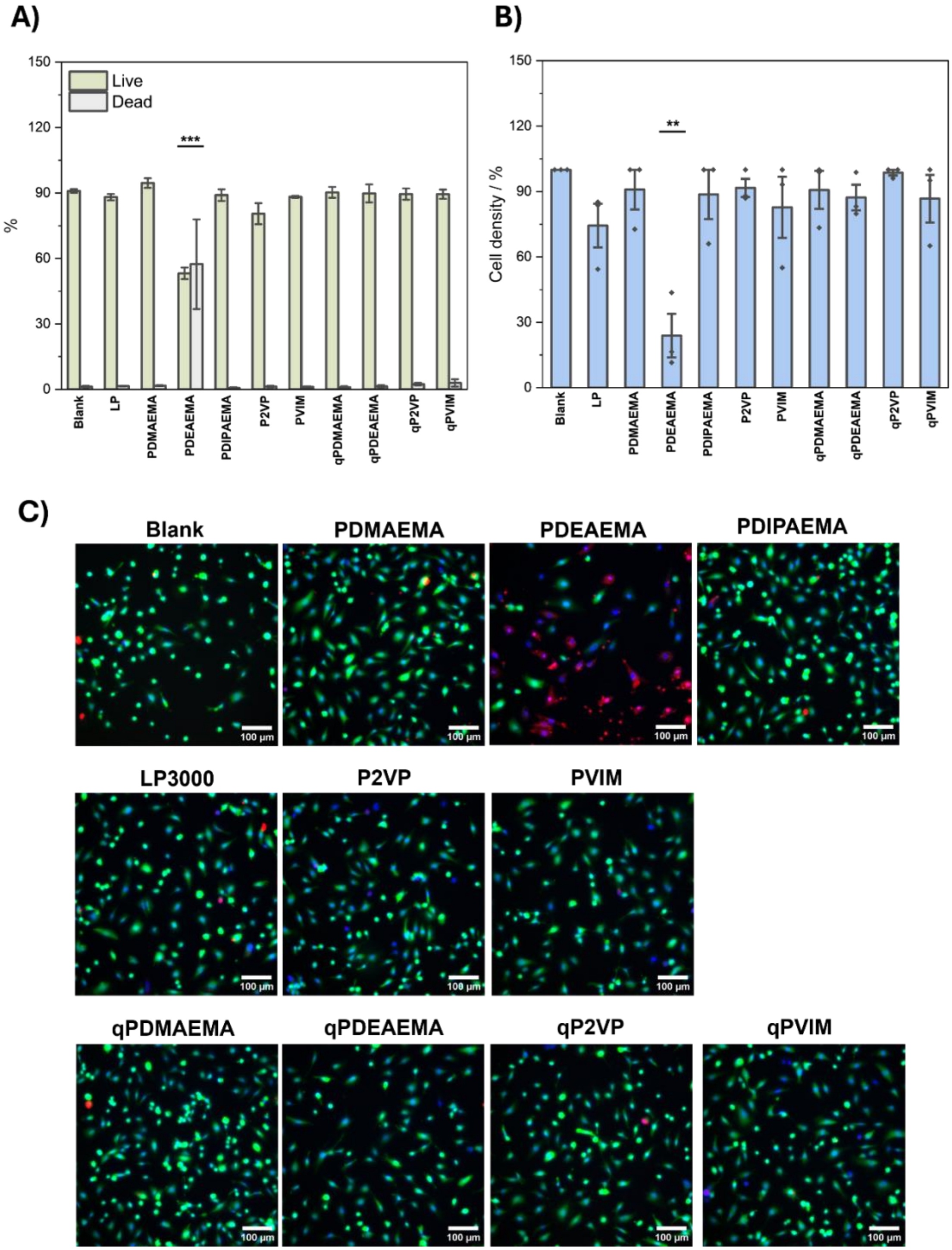

Figure S10. *Cytotoxicity studies of RNA/polymer brush-functionalised* SiO_2 *nanoparticles (300 nm core) complexes on HUVECs at day 1 post-transfection. Quantification of* ***A)*** *live and dead cells and* ***B)*** *cell density.* ***C)*** *Representative micrographs from live-dead assay. Error bars represent s.e.m.; N=3. Statistical significance compared to non-transfected control *p < 0.05; **p < 0.01; ***p < 0.001.*

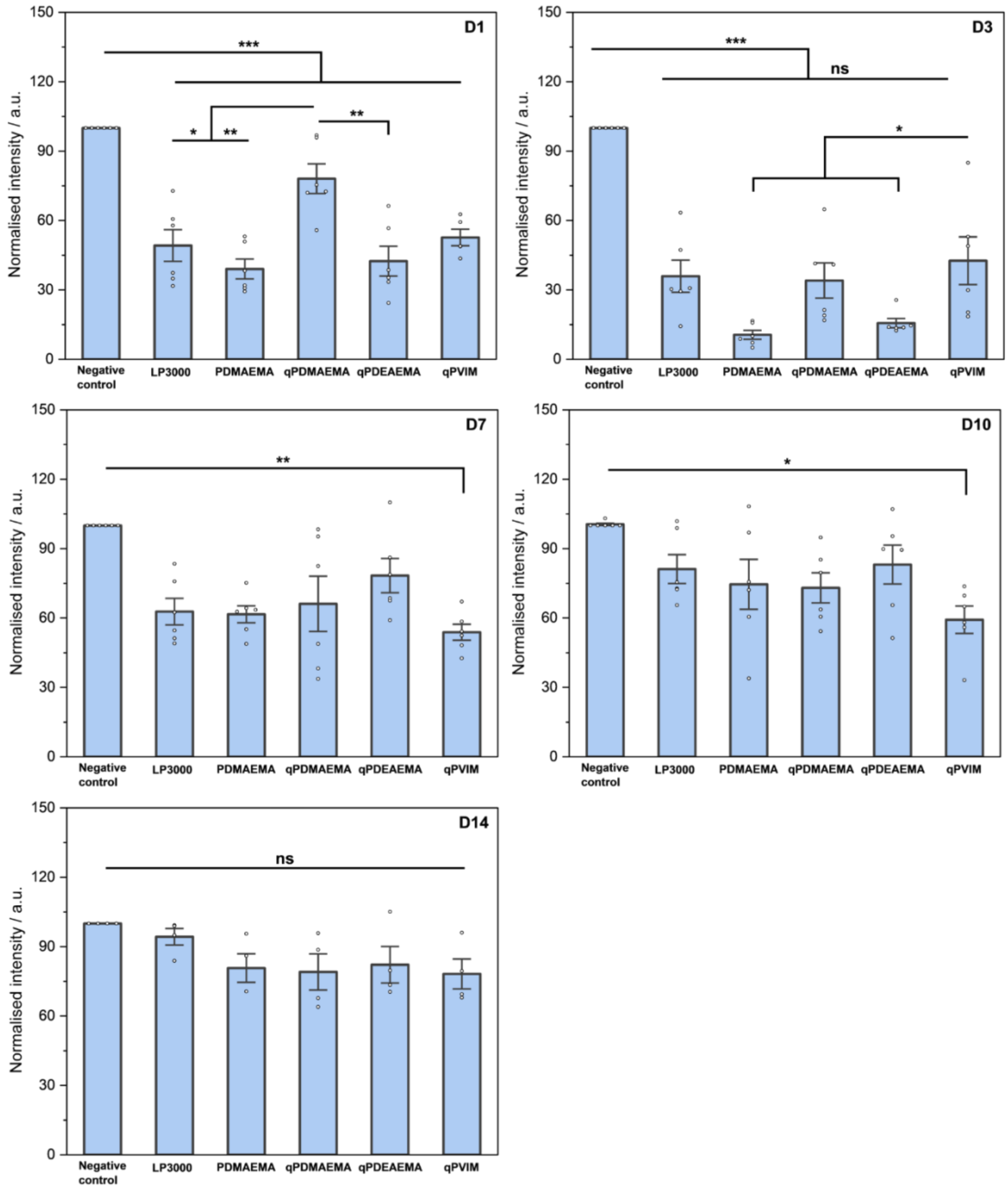

Figure S11. *Bar plots presenting the quantification of relative protein expression over 14 days post-transfection (D1-D14), normalised to LP3000 negative control. Error bars represent s.e.m.; N = 6 for D1-D10; N = 4 for D14. *p < 0.05; **p < 0.01; ***p < 0.001.*

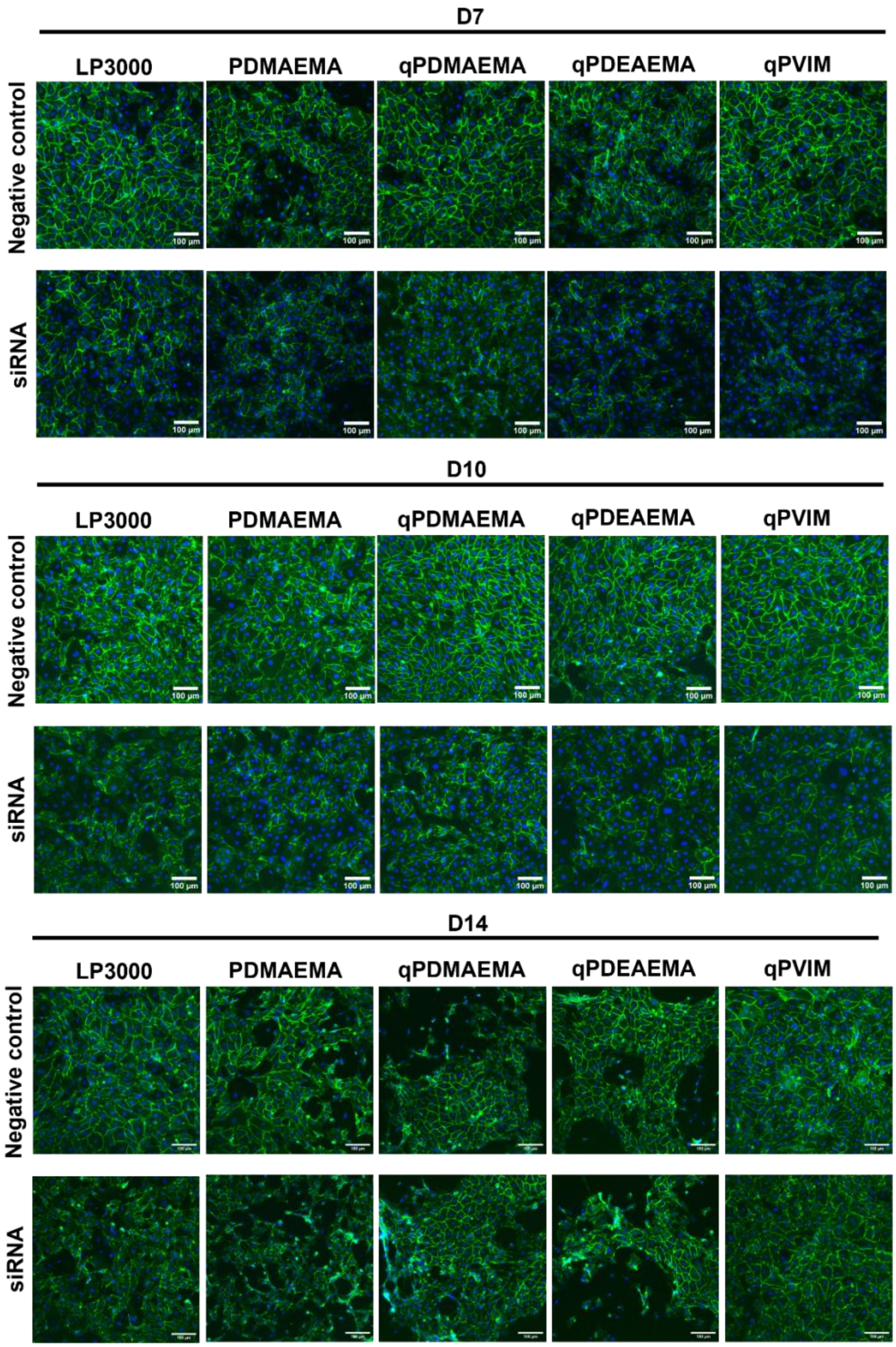

Figure S12. *Representative immunofluorescence images of long-term knockdown efficiency (7, 10 and 14 days) in HUVECs transfected with polymer brush-SiO_2 nanoparticles (300 nm core). Top row: negative controls (non-targeting siRNA); bottom rows: CD31-siRNA. CD31 expression (green) and nuclei (blue); scale bars: 100 µm.*

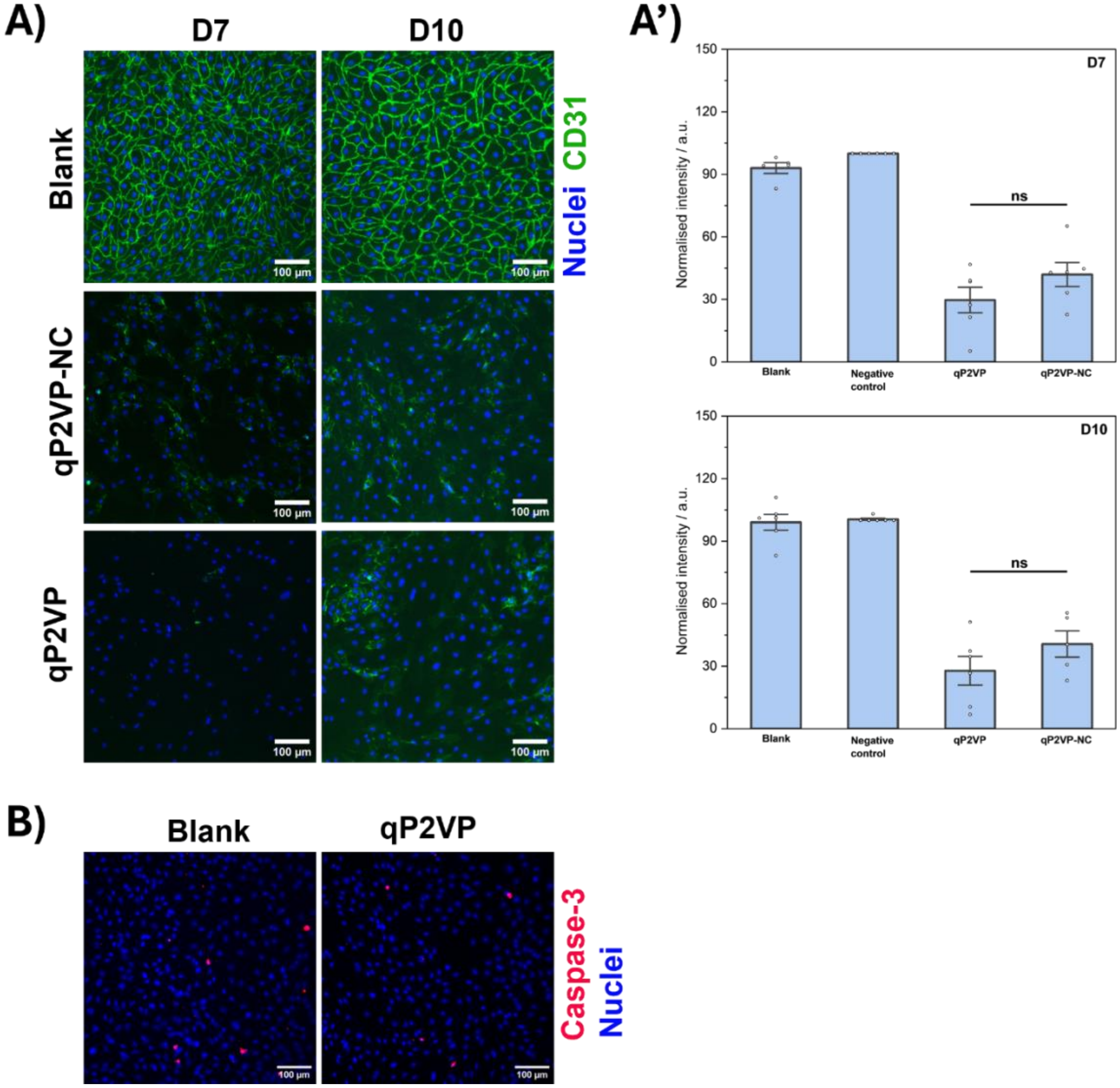

Figure S13. *Representative micrographs of* ***A-A')*** *CD31 staining and quantification of transfection of HUVECs with CD31-siRNA/qP2VP brush-functionalised SiO_2 nanoparticles (300 nm core) compared to non-targeting siRNA/qP2VP negative control qP2VP-NC) and blank (non-transfected control) at 7 and 10 days post-transfections.* ***B)*** *Caspase-3 staining for apoptotic cell death at 10 days post-transfection.*

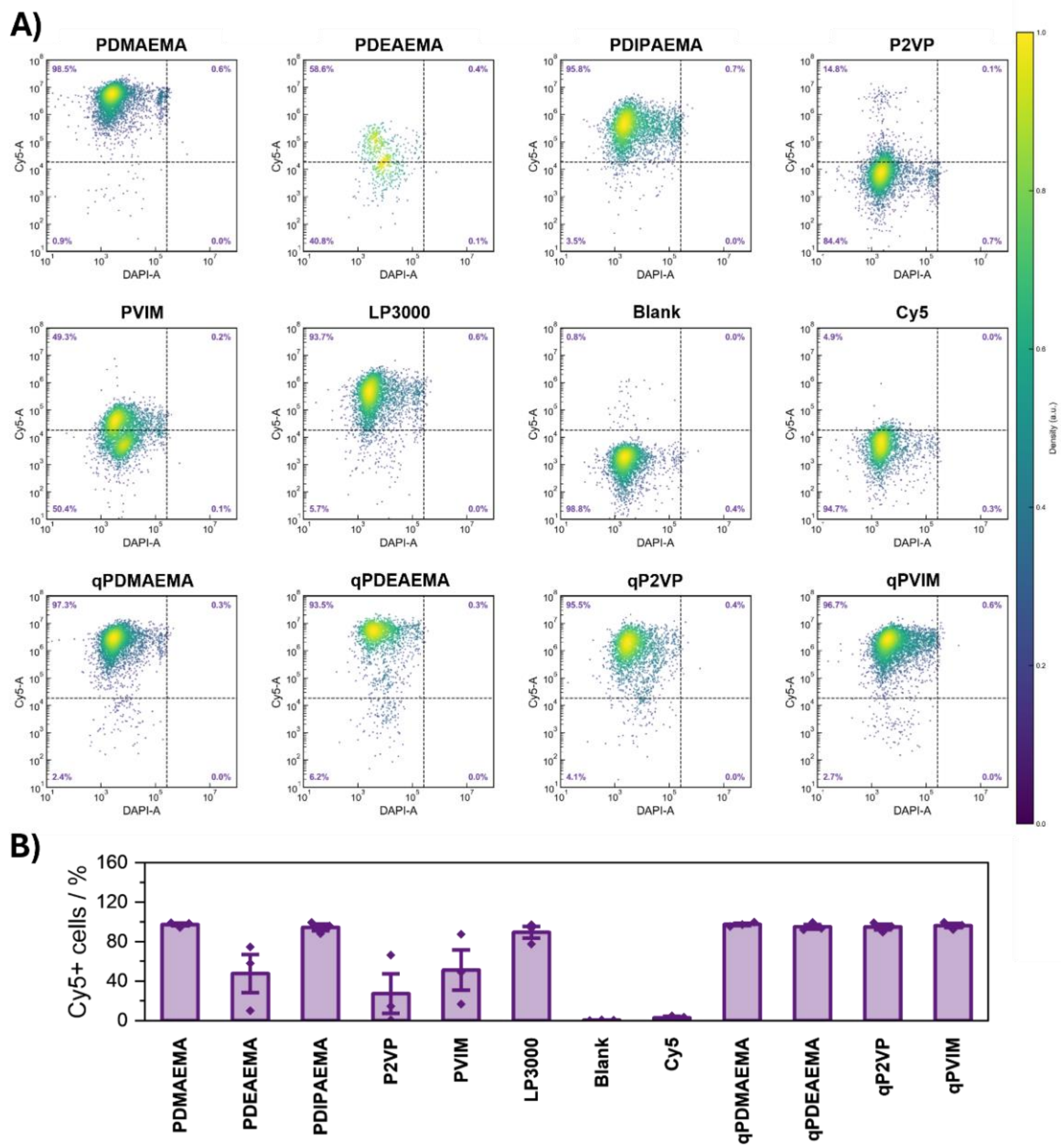

Figure S14. *Cellular internalisation of Cy5-tagged RNA, 24 h post-transfection in HUVECs transfected with polymer brush-functionalised* SiO_2 *nanoparticles (300 nm core), characterised by flow cytometry.* ***A)*** *Representative flow cytometry density plots (Cy5 versus DAPI staining) for all brush chemistries and controls, with quadrant percentages indicated. Cy5 is a free siRNA control without vector; Blank is a non-transfected control.* ***B)*** *Corresponding percentages of alive Cy5+cells (top left quadrants). Error bars represent s.e.m.; N = 3. For PDMAEMA, LP3000 and all quaternised chemistries, ≥ 90% of transfected cells internalised RNA.*

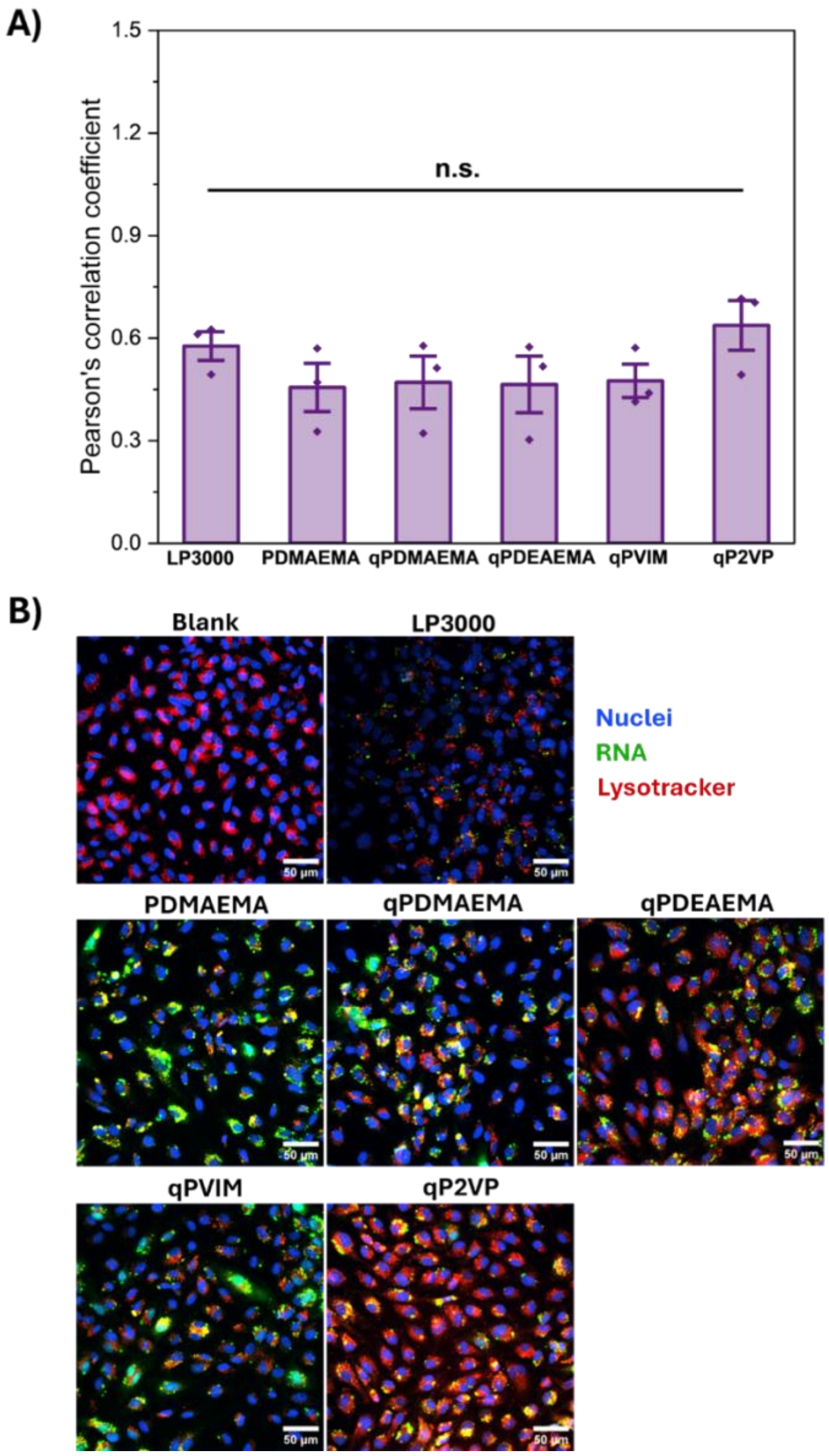

Figure S15. *RNA cellular internalisation studied with polymer brush-functionalised* SiO_2 *nanoparticles (300 nm core) 3 days post-transfection in HUVECs.* ***A)*** *Corresponding Pearson correlation coefficients for RNA and Lysotracker signals.* ***B)*** *Representative confocal micrographs. Error bars represent s.e.m.; N=3. No statistically significant differences were observed between treatments (*$p > 0.05$*).*
